# A multi-faceted study combining quantum chemical, Hammett parameters, and antibacterial assessment for imidazole: based green ionic liquids corrosion inhibition

**DOI:** 10.1186/s13065-026-01775-4

**Published:** 2026-04-14

**Authors:** Amira H. E. Moustafa, Hanaa H. Abdel-Rahman, Mohamed Hagar, Sherif A. A. Bishr

**Affiliations:** Chemistry Department, Faculty of Science, Alexandria University, Ibrahemia, P.O. 426, Alexandria, 21321 Egypt

**Keywords:** *N*-acetylimidazolium ionic liquids (ACIM), Carbon steel (CS), Hammett, MS, SEM/EDX, XPS, DFT/MEP/MD

## Abstract

Stringent environmental regulations drive the quest for green corrosion inhibitors (greenish), with ionic liquids being prime candidates due to their high stability and low volatility and non-flammability. The present work investigates the synthesis, characterization, and corrosion inhibition performance of green ionic liquids based (ILs) on imidazole. The newly synthesized ***1-acetyl-3-benzoyl-1H-imidazol-3-ium derivatives*** **(ACIM)**, were successfully characterized. Their structures were validated using Fourier transform infrared spectroscopy (***FT-IR***), ^1^H nuclear magnetic resonance (^***1***^***H NMR***), Carbon-13 (^***13***^***C NMR***) and Mass spectrometry **(MS)**. Their corrosion inhibition performance in 8 M H_3_PO_4_ was evaluated using Galvanostatic polarization and weight loss methods. We measured the limiting current value and the mass transfer rate which revealing excellent protection efficiency for **ACIM-OMe** (81.08% at 20.58 × 10⁻⁵ M, 298 K). Thermodynamic parameters confirmed spontaneous adsorption, evidenced by negative *ΔG*_*ads*_ values (− 29.77 to − 38.26 kJ mol⁻¹). Based on the typical energy range of − 20 to − 40 kJ mol⁻¹, the mechanism is interpreted as **mixed**, involving both physical (electrostatic) and chemical (coordinate bonding) interactions. This aligns with the *El-Awady’s* adsorption isotherm model. Scanning electron microscopy (SEM), energy-dispersive X-ray spectroscopy (EDX), and X-ray photoelectron spectroscopy (XPS) were employed. These techniques were used to analyze and visualize the modifications of the CSs’ surface following **ACIM** adsorption. Atomic absorption spectroscopy (AAS) confirmed the inhibitor’s effectiveness, showing a 77.17% efficiency for **ACIM-OMe** based on reduced Fe^2+^ ion concentration in solution. Antibacterial testing revealed that only **ACIM-H** showed measurable antibacterial activity against *Salmonella (positive inhibition zone)*, though it was less potent than the Kanamycin standard. Additionally, the substituent groups of **ACIM** derivatives can impact the inhibitors’ coordination ability and binding potential in the corrosion process. The influence of these substituent groups on the inhibition efficiency was quantitatively rationalized and correlated using *Hammett* (*σ*) and *Taft* dual parameter (σ*), respectively. Hammett analysis of the meta/para-substituted derivatives confirmed that inhibition efficiency increases with electron-donating ability, evidenced by a negative reaction constant (ρ = − 0.313). Complementing the experimental work, theoretical studies using molecular dynamics (**MD**) and detailed density functional theory (**DFT**) successfully modeled the adsorption and surface interactions of **ACIM** on CS. Finally, theoretical insights align with the experimental findings.

**Supplementary Information:**

The online version contains supplementary material available at 10.1186/s13065-026-01775-4.

## Introduction

Metal corrosion remains a serious and costly challenge worldwide, with annual expenses for equipment replacement, maintenance, and deterioration reaching billions of dollars. Among construction materials, carbon steel (CS) is widely employed due to its affordability and excellent mechanical properties [1]. However, its vulnerability to corrosive environments, particularly in acidic solutions, leads to significant economic losses. In phosphoric acid, frequently applied in pickling and descaling operations, CS deteriorates rapidly unless efficient protective strategies are adopted [2].

Several methods, including anodic and cathodic protection, protective coatings, and corrosion inhibitors, have been proposed to combat corrosion. Among these, the use of inhibitors has emerged as one of the most promising approaches, especially under harsh conditions. When selecting a corrosion inhibitor, key factors such as cost-effectiveness, environmental impact, and adsorption capacity must be taken into account. Carbon steel (CS) continues to be a preferred structural material in various industries including power generation, petroleum, construction, and household applications due to its low cost and high strength [3, 4]. However, its chemical instability in corrosive environments, particularly in acidic media, leads to rapid deterioration, posing significant economic and safety risks unless effective protective measures are implemented.

Organic corrosion inhibitors, particularly those containing heteroatoms such as nitrogen, sulfur, and oxygen, have attracted significant attention due to their ability to form coordinate bonds with the vacant d-orbitals of iron [5]. This interaction results in the formation of protective films that reduce corrosion rate. In addition, the presence of aromatic rings and polar groups enhances adsorption through electrostatic and π–d interactions [6], further strengthening their protective capacity. The efficiency of such inhibitors at low concentrations makes them attractive candidates for corrosion control [7].

Despite their effectiveness, many synthetic organic inhibitors pose challenges related to toxicity [8], bioaccumulation, and environmental persistence [9]. The harmful effects associated with their preparation, use, and disposal have motivated the search for sustainable alternatives. As a result, natural products and environmentally benign compounds have gained attention as safe and effective substitutes. These green inhibitors offer advantages such as low toxicity, biodegradability, and wide availability [2, 8]. With the global emphasis on sustainable development, the demand for eco-friendly corrosion protection strategies has become increasingly critical .

Ionic liquids (ILs), generally defined as salts with melting points below 100 °C, have recently been investigated as promising green corrosion inhibitors [10]. Their unique properties, including negligible vapor pressure, high thermal stability, tunable solubility, and non-flammability, make them attractive for various industrial applications [11]. Functionalization of ILs with nitrogen-containing cations, such as imidazolium [12], benzimidazolium [5], pyridinium [13], pyrrolidinium, and piperidinium [5], has demonstrated remarkable corrosion inhibition performance in acidic media [14]. Among these, imidazole-based ILs are particularly interesting because of their electron-rich aromatic rings, strong adsorption capabilities, and ability to form durable protective films on CS surfaces.

In addition to their primary role in corrosion mitigation, certain ionic liquids have shown potential antibacterial properties, which may extend their utility in environments prone to microbially influenced corrosion (MIC) a significant concern in sectors such as marine engineering, oil and gas, and water treatment. In such settings, microbial biofilms can accelerate metal degradation by creating localized electrochemical cells and corrosive metabolites. Inhibitors that combine corrosion protection with antibacterial activity could therefore offer dual-functional performance, addressing both chemical and biological degradation pathways. Although MIC is not the focus of the present acidic corrosion system, preliminary antibacterial assessment provides a broader understanding of the multifunctional potential of imidazolium-based ILs and supports their application in complex industrial environments where both abiotic and biotic corrosion mechanisms coexist [15].

The inhibitory efficiency of ILs is influenced by several factors, including the molecular structure, substituent groups, halide counterions, and the characteristics of the corrosive medium. Substituents play a critical role in modulating electron density at donor sites, thereby affecting adsorption strength and inhibition efficiency. Electron-donating groups (e.g., –NH_2_, –OH, –OCH_3_) increase electron density [16], enhancing adsorption, while electron-withdrawing groups (e.g., –NO_2_, –CN, –COOH) reduce it [17]. These effects are quantitatively described by Hammett constants (σ), which account for both inductive (I-) and resonance (R-)contributions. Consequently, understanding the substituent effect is essential for the rational design of highly efficient IL-based inhibitors [18].

Although several studies have explored ionic liquids as corrosion inhibitors, limited attention has been given to understanding the correlation between substituent effects and inhibition efficiency in phosphoric acid solutions. Furthermore, while experimental studies have demonstrated their potential, there remains a need to complement these findings with theoretical investigations, including density functional theory (DFT) [19] and molecular dynamics (MD) simulations [2, 20]. Such combined approaches provide deeper insight into adsorption mechanisms, structural orientation, and inhibitor metal interactions at the molecular level.

Despite the growing interest in ionic liquids (ILs) corrosion inhibitors, imidazole-based ionic liquids (ILs) are promising green corrosion inhibitors, key gaps persist in understanding their structure activity relationships in phosphoric acid, quantifying substituent effects, and exploring multifunctional traits like antibacterial activity. Prior research has focused mainly on hydrochloric or sulfuric acid, with limited systematic study in phosphoric acid. Although, it is less corrosive than other mineral acids and used in many manufacturing processes. Additionally, the integration of experimental methods with advanced theoretical tools such as Hammett analysis, molecular dynamics (MD), and density functional theory (DFT) to probe adsorption and substituent influence remains underdeveloped.

To bridge these gaps, this study aims to design ILs based on imidazole (**ACIM derivatives**) as green corrosion inhibitors for CS in phosphoric acid. Investigating the impact on electrochemical corrosion processes and their effect on the mass transfer rate from the CS surface to the bulk solution. The mass transfer coefficient can be found using the limiting current method, which has gained popularity in recent years. Study their performance using surface analysis techniques SEM, EDX, XPS, and AAS. In addition to evaluating corrosion inhibition performance, preliminary antibacterial screening was conducted on the synthesized ILs to assess their potential multifunctional utility in environments where microbial activity may contribute to material degradation (e.g., microbiologically influenced corrosion). Although secondary to the primary corrosion focus, this screening provides insight into the broader applicability and bio-interaction profile of the **ACIM** compounds. Further investigation and discussion of global quantum chemical descriptors, Fukui indices, the results of density functional theory (DFT), and experimental data are validated through molecular dynamics simulation to gain a better understanding of the mechanism, interactions, and structural orientation of the four inhibitors and the CS metallic surface. By integrating substituent analysis with theoretical and experimental approaches, this study aims to provide comprehensive insights into the structure performance relationships of imidazole-based ILs as green corrosion inhibitors.

## Experimental section

### Design and production of corrosion inhibitors

Without additional purification, all the reagents and solvents used were of the highest analytical reagent grade. We bought fine chemicals and solvents from Sigma-Aldrich and BDH Chemicals Ltd. A Stuart Scientific SMP1 was used to measure melting points. UV fluorescent Silica gel Merck 60 F254 plates were used for thin layer chromatography (TLC), and a UV lamp (254 nm) was used to view the spots. A Perkin-Elmer 1430 series Fourier transform infrared (FT-IR) spectrometer was used to perform FT-IR spectroscopy. Using tetramethylsilane **(**TMS) as an internal reference, ^1^H, ^13^C nuclear magnetic resonance (^1^H NMR) and (^13^C NMR) spectra were acquired using a Bruker spectrometer (400 MHz). Mass spectrometry analysis was conducted using an Agilent 6500 series Q-TOF mass spectrometer. The instrument featured an electrospray ionization (ESI) source and was operated in positive ion mode. Data collection covered a mass range extending up to 3200 m/z. All operations were managed through Agilent MassHunter software (Version B.09.00).

**Fig. 1:**
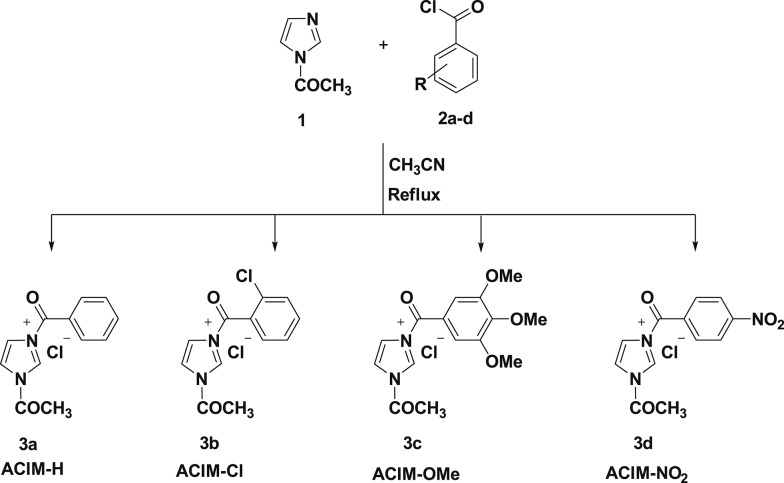
scheme for Synthesis of imidazolium-based ionic liquids **3a-d**

TLC confirmed that the reaction was complete after a mixture of *N*-acetylimidazole (***1***) (1 mmol) and the corresponding benzoyl chloride derivatives ***2a-d*** (1 mmol) dissolved in acetonitrile (20 mL) was heated under reflux for 6–8 h. The desired imidazolium ionic liquids ***3a–d*** were obtained by filtering and/or extracting the resultant product using chloroform. They are typically designed and developed using nitrogen-containing molecules by quaternizing their nitrogen atoms. In this study, the desired imidazolium-based ionic liquids **3a-d** (Fig. 1) were synthesized by initiating the reaction with *N*-acetylimidazole (**1**). Thus, the desired *N*-acetylimidazolium ionic liquids **3a-d** in 91–94% were obtained by thermally alkylating *N*-acetylimidazole (**1**) with a few benzoyl chloride derivatives **2a-d** in acetonitrile. The efficacy of this quaternization method was validated by the spectroscopic data of the resulting ILs **3a-d**. The addition of the phenyl group of the used benzoyl derivatives to the quaternization reaction of the N-imidazole atom was thus confirmed by the proton NMR spectra, which showed the existence of additional aromatic protons ranging from δ_H_ 7.19 ppm to 8.37 ppm. The methyl protons of the acetyl group were identified as the source of the distinct singlet between δ_H_ 2.41–2.52 ppm, whereas the methyl protons of the methoxy groups for the IL **3c** were identified as the source of the singlets resonating at δ_H_ 3.68–3.78 ppm. All the spectrum data described in the Sup. Figures (1–12).

### Testing medium preparation and electrode sample

Fisher Chemicals Ltd. provided analytical grade phosphoric acid (85% w/w) for the preparation of the corrosive medium, 8 M H_3_PO_4_ [2, 21]. CS with a surface area of 10 cm^2^ served as the electrode sample for the electrochemical experiments, and the JEOL apparatus JSM-IT200 model was used to determine the composition (wt%): Carbon (0.14%), silicon (0.02%), manganese (0.56%), phosphorus (0.03%), nickel (0.01%), chrome (0.01%), vanadium (0.01%), aluminium (0.03%), sulphur (0.04%), and iron (99.15%). We must understand that the 8 M H_3_PO_4_ was chosen because the mass transport concept can be used to analyze the effect of the H_3_PO_4_ concentration on the value of the limiting current, I_Lim_ [19, 22].

100 mL of the produced phosphoric acid was used in each experiment, along with different concentrations of the investigated IL inhibitors (2.94, 5.88, 8.82, 14.70, and 20.58 × 10^− 5^) M, which were dissolved in water for **ACIM-OMe**,** ACIM-H**,** ACIM-Cl**, and **ACIM-NO**_**2**_. For them, the I_Lim_ was recorded at several temperatures: 298 K, 303 K, 308 K, and 318 K.

### Galvanostatic of anodic polarization

The Galvanostatic approach is a popular method for assessing the inhibition efficiency of inhibitors due to its precision, simplicity, and straightforward application. The polarization experiments in this investigation were conducted using the standard procedures previously stated [19, 23]. Steady-state conditions were ensured by a 30 min stabilization, followed by a slow potential sweep (0.5 mV/s). Triplicate measurements showed excellent reproducibility (± 2%), confirming that the adsorbed inhibitor layer reached a stable configuration during the test.The final corrosion data represent the average of three independent measurements.

### Weight loss measurements

The corrosion examination was conducted using rectangular CS specimens that measured 2 cm by 2 cm by 0.3 cm [23]. With and without different inhibitor dosages, the weighted samples were submerged in a 250 mL beaker filled with 100 mL of an 8 M H_3_PO_4_ solution. Temperature, inhibitor concentration, and immersion duration were among the crucial experimental variables that were meticulously regulated. Following the specified exposure time, the samples were taken out, cleaned with distilled water, dried, and weighed again. Weight loss was calculated as the mass difference before and after immersion for a period time ranging from ½ hour to 24-hour immersion period at 298 K for all **ACIM** derivatives and so, directly measure the corrosion rate. This reliable method assesses inhibitor performance by reflecting real material degradation under controlled conditions. Lower weight loss indicates higher inhibitor efficiency, signifying the formation of a protective layer on the CS surface. Each experiment was conducted in triplicate to ensure reproducibility, with average weight loss reported to minimize experimental errors. Quantitative insights into the inhibitors’ protective processes were obtained by calculating the thickness loss and inhibitory efficiency (%*I*_*Eff*_) using the weight loss data [24].

### Analysis of spectroscopy

To determine the amount of iron ions released into the solution during corrosion studies and for environmental protection, the *Fe*^*2+*^ concentration was measured using atomic absorption spectroscopy (AAS) and approximated using the Analytik Jena Contraa 300 AAS.

### Surface attributes

The shielding layer on the CS surface, along with its morphological description, is visible using a scanning electron microscope (SEM). Using a JEOL apparatus, the JSM-IT200 model was combined with an electron dispersive X-ray spectroscopy (EDX) analyzer to identify the elemental components of the layers that developed on the corroded surface.

K-ALPHA (Thermo Fisher Scientific, USA) conducted X-ray photoelectron spectroscopy (XPS) investigations using monochromatic X-ray AL K-α radiation (energy: 10 to 1,350 eV) in a vacuum of 10^−^9 with full-spectrum pass energy of 200 eV and narrow-spectrum pass energy of 50 eV. The diameter of the analysis point was 400 μm. Every binding energy value for the C1s line originating from adventitious carbon was established.

### Hammett equation

The Hammett equation serves as a valuable framework for describing how the electronic effects of substituents influence both the rate and equilibrium of organic reactions. Beyond its traditional applications in organic chemistry, this equation has been extensively investigated and employed to characterize the interactions between metallic substrates, such as CS surfaces, and organic inhibitors, including ionic liquid inhibitors. Furthermore, it provides insight into how various substituents in organic compounds can contribute to corrosion inhibition. The Hammett equation is presented as follows [18]:

1 $$log\frac{{{K_R}}}{{{K_H}}}=~\rho \sigma \,\left( {{\text{original Hammett equation}}} \right)$$

2 $$log\frac{{1 - \% I{E_R}}}{{1 - \% I{E_H}}}=~\rho \sigma \,\left( {{\text{simple Hammett equation with }}\% {\mathrm{IE}}} \right)$$

where *K*,* %IE*, *σ* $and\rho$are the equilibrium constants of the adsorption-desorption process (*K*_*R*_ with substituent and *K*_*H*_ without substituent), inhibitory efficiency, Hammett constant, and the value of reaction parameters respectively.

### Antibacterial activity

The antibacterial effectiveness of each inhibitor against pathogenic strains like Salmonella spp. was assessed using the Agar disc diffusion assay. The strains were obtained from the Microbiological Resources Center (MERCIN), Faculty of Agriculture, Ain Shams University, Cairo, Egypt, and preserved at -80 °C to maintain viability. Before testing, the strain was cultivated in nutrient broth at 37 °C for 24 h. Mueller-Hinton agar plates inoculated with the bacterial suspension were then covered with sterile filter paper discs impregnated with the inhibitors. Afterward, the plates were incubated at 37 °C for 18 to 24 h to promote bacterial growth and inhibitor diffusion [25]. The antibacterial activity of the discs was assessed by measuring the zones of inhibition (ZOI) after incubation. Larger inhibition zones indicated greater antimicrobial potency, demonstrating the inhibitor’s ability to stop bacterial growth. This approach is a popular and standardized method for evaluating antibiotic efficacy due to its quick and repeatable results. The study also considered variables like disc concentration, diffusion rate, and bacterial susceptibility to ensure accurate data interpretation [26].

### Theoretical studies

Theoretical calculations were performed using density functional theory (DFT) at the B3LYP/6-311G(d, p) level employing the Gaussian 09 software package to obtain optimized geometries and electronic properties of the investigated inhibitors in both gas and aqueous phases. The output data were further analyzed using GaussSum software to extract and visualize key quantum chemical parameters. Several descriptors were evaluated, including electronegativity, work function, chemical hardness, softness, electron localization function (ELF), molecular electrostatic potential (MEP), Fukui functions, and local dual descriptors, in order to interpret the experimental observations. In addition, molecular dynamics (MD) simulations were carried out using the DMol³ module within Materials Studio with appropriate control parameters to gain further insight into the adsorption behavior and inhibition mechanism of the imidazolium-functionalized ionic liquids on the CS surface.

## Results and discussion

### Galvanistatic polarization study

To understand the kinetics and suppression mechanism of the investigated **ACIM** inhibitors at the CS/H_3_PO_4_ interface, electrochemical polarization studies were conducted. The analysis of current-potential curves derived from galvanostatic measurements served this purpose.

**Fig. 2:**
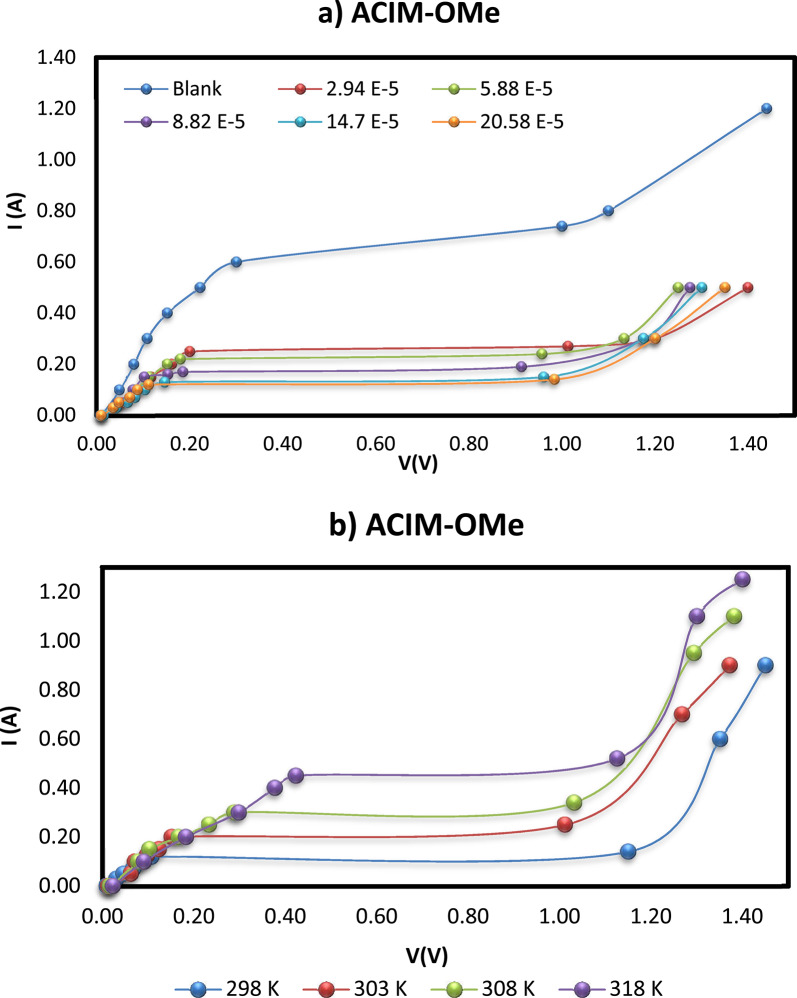
Polarization curve of the tested CS: (**a**) at different concentrations at 298 K & (**b**) at different temperatures for concentration 20.58 × 10^− 5^ M

Table 1 shows that, in comparison to the blank curve, the addition of **ACIM** derivatives significantly decreased the corrosion limiting current values by raising inhibitor concentrations and enhancing them with temperature. **ACIM** inhibitors restrict the CS surface’s active sites, making it more difficult for the CS electrode to corrode after their adsorption, and thereby demonstrating exceptional corrosion resistance. The following Eq. (3) was used to calculate the inhibitory efficiency (%*I*_*Eff*_) values and the extent of surface coverage (θ) [2, 19]:

3 $$\% {I_{Eff}}=\frac{{{I_{Lim}}\left( {{\mathrm{blank}}} \right)~ - {\text{}}{I_{Lim}} {{\left( {\mathrm{ACIM}} \right)}}}}{{{I_{Lim}}\left( {{\mathrm{blank}}} \right)}} \times 100$$

where the limiting currents without and with an inhibitor concentration are denoted by *I*_*Lim*_ (blank) and *I*_*Lim*_(**ACIM**), respectively, the % I_Eff_ **ACIM** increased as the inhibitor concentration increased, reaching 81.08% for 20.58 × 10^− 5^ M of ACIM-OMe, which is the most effective inhibitor, as shown in Table 1. It should be noted that the inhibitor would be desorbed from the CS surface if the concentration exceeded 20.58 × 10^− 5^ M, as shown in Fig. 2a for **ACIM-OMe** as an example, and the data for other inhibitors are provided in the Sup. Figures (13–18). However, in the case of temperature change, the I_Eff_ of **ACIM** decreases from 81.08% to 61.19% due to the desorption of inhibitor molecules at higher temperatures [27]. This can be attributed to the high rates of CS dissolution at high temperatures, which are caused by increased solution agitation resulting from the rapid development of H_2_ gas.

Additionally, this might make it harder for inhibitors to adsorb on the CS surface. The tabulated results confirm that the tested molecules act through adsorption onto the CS surface, promoting the formation of a protective inhibitory film that functions as a barrier between the corrosive medium and the steel substrate. Among the inhibitors studied, **ACIM-OMe** exhibited the highest inhibition efficiency, suggesting lower desorption and stronger film formation, thereby making it the most effective inhibitor (Fig. 2b).

**Table 1:** At varying temperatures and concentrations, limiting current (I_lim_) levels and inhibitory efficiency (%${I}_{Eff}$) with and without **ACIM**

ILs	C x 10^5^ (M)	298 K		303 K		308 K		318 K	
		I_Lim_	% I_Eff_	I_lim_	% I_Eff_	I_lim_	% I_Eff_	I_lim_	% I_Eff_
ACIM-OMe	Blank	0.74	0.00	0.89	0.00	1.07	0.00	1.34	0.00
	2.94	0.27	63.51	0.48	46.07	0.60	43.92	0.86	35.82
	5.88	0.24	67.57	0.40	55.06	0.51	52.34	0.80	40.30
	8.82	0.19	74.32	0.32	64.04	0.40	62.62	0.67	50.00
	14.70	0.15	79.73	0.29	67.42	0.38	64.49	0.54	59.70
	20.58	0.14	**81.08**	0.25	**71.91**	0.34	**68.22**	0.52	**61.19**
ACIM-H	Blank	0.74	0.00	0.89	0.00	1.07	0.00	1.34	0.00
	2.94	0.40	45.95	0.58	34.83	0.72	32.71	1.06	20.89
	5.88	0.35	52.70	0.53	40.45	0.66	38.32	1.00	25.37
	8.82	0.32	56.76	0.45	49.44	0.58	45.79	0.91	32.09
	14.70	0.29	60.81	0.44	50.56	0.56	47.66	0.87	35.07
	20.58	0.28	**62.16**	0.43	**51.68**	0.55	**48.60**	0.86	**35.82**
ACIM-Cl	Blank	0.74	0.00	0.89	0.00	1.07	0.00	1.34	0.00
	2.94	0.49	33.78	0.65	26.97	0.81	24.30	1.13	15.67
	5.88	0.41	44.59	0.59	33.71	0.72	32.71	1.01	24.63
	8.82	0.33	55.40	0.48	46.07	0.59	44.86	0.93	30.60
	14.70	0.31	58.11	0.47	47.19	0.57	46.73	0.89	33.58
	20.58	0.30	**59.46**	0.46	**48.31**	0.56	**47.66**	0.88	**34.33**
ACIM-NO_2_	Blank	0.74	0.00	0.89	0.00	1.07	0.00	1.34	0.00
	2.94	0.54	27.03	0.70	21.35	0.87	18.69	1.21	9.70
	5.88	0.45	39.19	0.63	29.21	0.77	28.04	1.11	17.16
	8.82	0.42	43.24	0.56	37.08	0.68	36.45	1.01	24.63
	14.70	0.40	45.94	0.54	39.32	0.67	37.38	0.99	26.12
	20.58	0.39	**47.30**	0.53	**40.45**	0.66	**38.32**	0.95	**29.10**

### Isotherm modeling and thermodynamic parameter evaluation

Understanding the adsorption process through an appropriate adsorption isotherm is highly valuable, as it provides deeper insight into the interaction between the inhibitor molecules and the CS surface, and consequently into the overall corrosion control mechanism. To identify the most suitable adsorption isotherm model, the degree of surface coverage (θ) of the CS surface was evaluated. An increase in θ with rising inhibitor concentration indicated the progressive formation of a protective film over the CS surface, which in turn decreased the available active sites for corrosive ion attack, thereby mitigating corrosion [28].

The linear forms of several adsorption isotherm models were considered for the investigated inhibitors: *Langmuir*,* Frumkin*, and *El-Awady*, all evaluated at 298 K with varying inhibitor concentrations, as presented in Table 2.

**Table 2:** Linear isotherm equations for **ACIM** derivatives at 298 K

Isotherm model	Linear form	Plot
Langmuir	$\frac{\mathrm{C}}{{\uptheta}}=\frac{1}{{\mathrm{K}}_{\mathrm{a}\mathrm{d}\mathrm{s}}}+\mathrm{C}$	$\frac{\mathrm{C}}{{\uptheta}}$vs. C
Frumkin	$\mathrm{l}\mathrm{o}\mathrm{g}\left[\mathrm{C}\right(\frac{{\uptheta}}{1-{\uptheta}}\left)\right]=2.303\mathrm{l}\mathrm{o}\mathrm{g}{\mathrm{K}}_{\mathrm{a}\mathrm{d}\mathrm{s}}+2\mathrm{a}{\uptheta}$	$\mathrm{L}\mathrm{o}\mathrm{g}\left[\mathrm{C}\right(\frac{{\uptheta}}{1-{\uptheta}}\left)\right]\mathrm{v}\mathrm{s}{\uptheta}$
El-Awady	$\mathrm{l}\mathrm{o}\mathrm{g}\left(\frac{{\uptheta}}{1-{\uptheta}}\right)=\mathrm{l}\mathrm{o}\mathrm{g}{\mathrm{K}}^{{\prime}}+\mathrm{y}\mathrm{l}\mathrm{o}\mathrm{g}\mathrm{C}$ $(\mathrm{K}={\mathrm{K}{\prime}}^{1/y})$	$\mathrm{l}\mathrm{o}\mathrm{g}\left(\frac{{\uptheta}}{1-{\uptheta}}\right)\mathrm{v}\mathrm{s}\mathrm{l}\mathrm{o}\mathrm{g}\mathrm{C}$

where *θ* = surface coverage = *I*_*Eff*_*%*, *C* is the concentration of inhibitors, *a* is the interaction in the adsorbed film layer that is described by the phrase “lateral interaction.“, and *K*_*ads*_ is the temperature-dependent constant of equilibrium of the adsorption interaction [29].

**Fig. 3:**
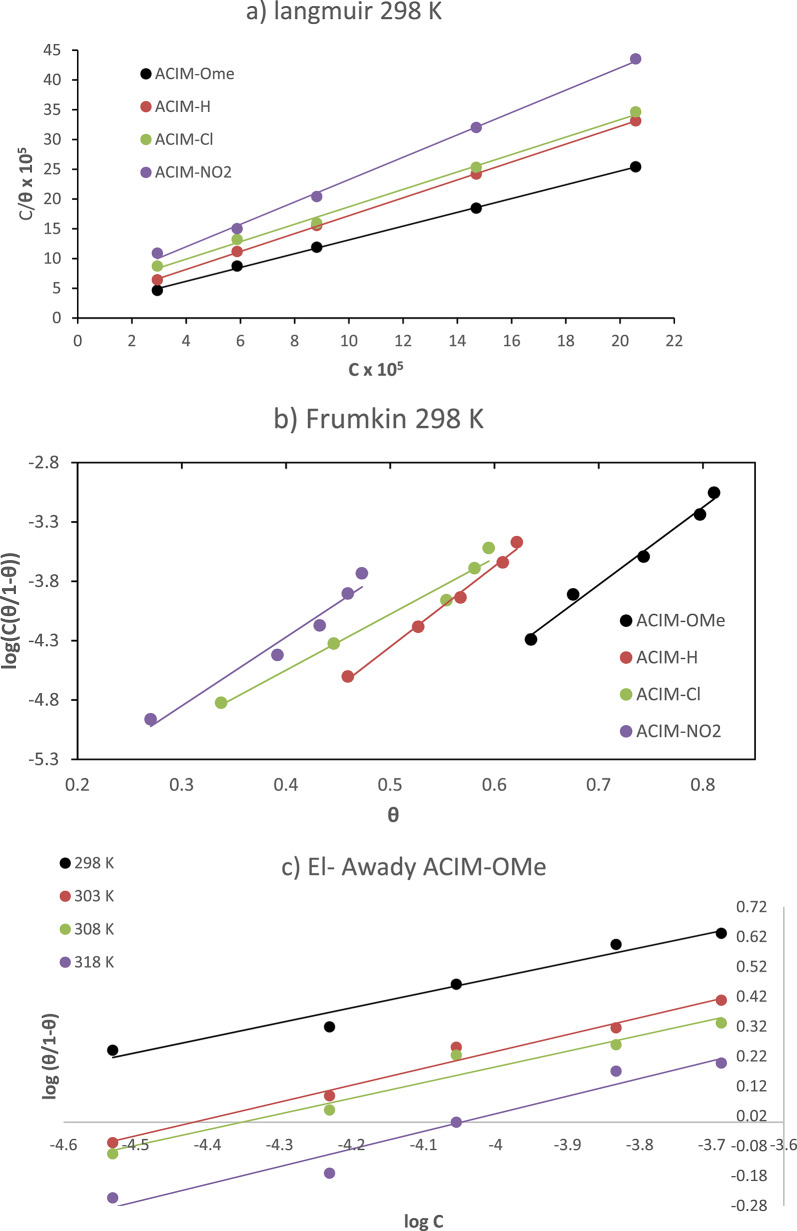
CS tested electrodes in 8M H_3_PO_4_ with varying concentrations at 298 K for **ACIM** adsorption isotherm models for isotherm equations in Table. 2** a** Langmuir, **b** Frumkin and for different temperatures **c** El-Awady for **ACIM-OMe**

Figure 3a; Table 3 show *Langmuir’s* adsorption relationship and isotherm variables for generated inhibitors. In this case, the linear correlation coefficient (*R*^*2*^) values are approaching one. According to the results, the *Langmuir* model exhibits the best linear connection; nonetheless, its slope deviates from unity, which can be attributed to intermolecular interactions, either attractive or repulsive, between adjacent adsorbed molecules [30].

**Table 3:** Adsorption parameters for *Langmuir*,* Frumkin*, and *El-Awady* at 298 K

Models parameters at 298 K										
ILs	Langmiur		Frumkin				El-Awady			
	slope	R^2^	R^2^	a	K_ads_	∆G_ads_ (kJ/mol)	R^2^	1/y	K_ads_	∆G_ads_ (kJ/mol)
**ACIM-OMe**	1.1587	0.9991	0.9861	3.2658	0.0002	10.8627	0.9668	1.99	91819.19	-38.26
**ACIM-H**	1.5041	0.9998	0.9921	3.3922	0.0004	9.2342	0.9879	2.88	22725.05	-34.80
**ACIM-Cl**	1.4635	0.9959	0.9709	2.3585	0.0002	11.6969	0.9278	1.78	11831.53	-33.19
**ACIM-NO**_**2**_	1.8796	0.9977	0.9613	2.8955	0.0001	12.0717	0.8965	2.26	4779.83	-30.94

For the CS electrode, the *Frumkin* adsorption isotherm is *Log[C (*$\frac{\theta}{1-\theta}\left)\right]$ *vs. θ* at 298 K in Fig. 3b. A linear correlation coefficient (*R*^*2*^) with slope (*2a*) and intercept ($2.303\mathrm{l}\mathrm{o}\mathrm{g}{\mathrm{K}}_{\mathrm{a}\mathrm{d}\mathrm{s}})$ was computed from the data presented in Table 3. The calculated values of *a* indicate positive lateral interactions, reflecting strong intermolecular interactions within the adsorbed layer. However, this observation contrasts with the relatively low equilibrium constant (*K*_*ads*_), suggesting that despite the high degree of interaction among adsorbed molecules, the overall adsorption affinity remains modest [28].

With a higher relationship coefficient of *R*^*2*^, *El-Awady’s* adsorption isotherm plot of *(log*$\frac{\theta}{1-\theta}$*)* as a component of (*logC*) is flawlessly direct, for example, Fig. 3c for **ACIM-OMe** and the rest of the Figures as a Sup. Figures (19–21). Lateral interactions between molecules that have been adsorbed on the outermost layer of CS are represented by the values of (*1/Y*). These findings confirm that the inhibitor molecules form a relatively thick adsorbed layer and engaging more than one active site on the CS substrate [2, 31].

Furthermore, the significant adsorptive capacity of inhibitors on the CS surface is indicated by the substantial value of *K*_*ads*_. The varying values of *K*_*ads*_ obtained at various solution temperatures are displayed in Table 4. Strong interactions between the inhibitory molecules and the CS may have been involved at this temperature, as indicated by the high values of *K*_*ads*_ seen at low temperatures. An increase in temperature results in a decrease in the *K*_*ads*_ value owing to the inhibitor molecule’s desorption. However, **ACIM-OMe** has the highest *K*_*ads*_ value, suggesting that it is the most potent inhibitor.

On the other hand, by analyzing corrosion inhibitors, thermodynamic parameters such as *ΔG*_*ads*_, *ΔH*_*ads*_, and *ΔS*_*ads*_ provide essential insights into the nature and spontaneity of the adsorption process. We can see how *K*_*ads*_ and the free energy of adsorption (*ΔG*_*ads*_) relate to one another by Eq. 4, where *R*, *T*, and 55.5 represent the ideal gas constant, work temperature (298–318 K), and the concentration of liquid water in (g/L) at the CS/solution interface, respectively [32].

Table 4 presents the Gibbs’ free energy values for *El-Awady’s* isotherm at all considered temperatures. The values varied from − 29.77 KJ/mol to − 38.26 KJ/mol. This indicates the spontaneous adherence of the inhibitor molecules to the surface of the CS. Plotting a relationship between the *∆G*_*ads*_ and *T* yields the enthalpy (*∆H*_*ads*_) and entropy (*∆S*_*ads*_) of adsorption, two other crucial thermodynamic parameters. The computed values are shown in Table 4, derived from Eq. 5 and Sup. Figure 22, where *∆S*_*ads*_ is the negative value of the slope and *∆H*_*ads*_ is the intercept, both of which have a single value for each **ACIM** inhibitor [33].

4 $$\Delta {{\mathrm{G}}_{ads}}= - {\text{RTln }}\left( {{\mathrm{55}}.{\text{5 K}}} \right)$$

5 $$\Delta {G_{ads}}=\Delta {H_{ads}} - {\mathrm{T}}\Delta {S_{ads}}$$

**Table 4:** Thermodynamic parameters of **ACIM** derivatives as determined by *El-Awady* model

ILs	ACIM-OMe								ACIM-H							
T	*R* ^2^	y	1/y	K’	K_ads_	∆G_ads_ (kJ/mol)	∆H_ads_ (KJ/mol)	∆S_ads_ (J/mol.K)	*R* ^2^	y	1/y	K’	K_ads_	∆G_ads_ (kJ/mol)	∆H_ads_ (KJ/mol)	∆S_ads_ (J/mol.K)
298	0.9668	0.50	1.99	309.31	91819.19	-38.26	-74.45	124.30	0.9879	0.35	2.88	32.39	22725.05	-34.80	-103.99	233.80
303	0.9817	0.57	1.77	314.12	26233.97	-35.75			0.9077	0.38	2.65	28.44	7073.20	-32.45		
308	0.955	0.53	1.90	193.78	22522.50	-35.95			0.9304	0.36	2.77	21.63	5036.91	-32.11		
318	0.9556	0.59	1.70	243.56	11181.90	-35.26			0.9484	0.41	2.43	19.62	1400.45	-29.77		

ILs	ACIM-Cl								ACIM-NO_2_							
T	R^**2**^	y	1/y	K’	K_**ads**_	∆G_**ads**_ (kJ/mol)	∆H_**ads**_ (KJ/mol)	∆S_**ads**_ (J/mol.K)	R^**2**^	y	1/y	K’	K_**ads**_	∆G_**ads**_ (kJ/mol)	∆H_**ads**_ (KJ/mol)	∆S_**ads**_ (J/mol.K)
298	0.9278	0.56	1.78	195.84	11831.53	-33.19	-68.25	118.50	0.8965	0.44	2.26	42.30	4779.83	-30.94	-40.28	32.40
303	0.8908	0.51	1.96	79.09	5305.01	-31.72			0.926	0.49	2.06	46.49	2718.92	-30.04		
308	0.9028	0.57	1.77	126.88	5203.00	-32.20			0.8806	0.52	1.93	57.84	2526.89	-30.35		
318	0.9177	0.54	1.86	56.78	1847.85	-30.51			0.9249	0.68	1.46	153.00	1571.60	-30.08		

Physisorption is associated with *∆G*_*ads*_ values of ≤ 20 kJ/mol, whereas chemisorption corresponds to *∆G*_*ads*_ values of ≥ 40 kJ/mol. If *∆G*_*ads*_ is between − 40 and − 20 kJ/mol [2], adsorption would be mixed between chemical and physical adsorption (physicochemical). Based on the relatively high *ΔG*_*ads*_ values indicate that the inhibitors adsorb onto the metallic surface predominantly through a mixed (physicochemical) mechanism. The inhibitor’s adsorption is an exothermic interaction, as indicated by the negative value of *∆H*_*ads*_. This result may explain why the adsorption remains constant and the inhibition subsequently decreases as the temperature rises. The highest adsorption entropy positivity values (*∆S*_*ads*_) found in this work indicate an increase in disorder, which may result in the displacement of ions or water molecules from the surface [34, 35].

### Kinetic parameters

Another valuable approach for assessing corrosion resistance was the kinetic model, which provided more profound insights into the characteristics of the inhibitors. The Arrhenius Equation in logarithmic structure with temperatures ranging from 298 to 318 K is used to calculate the activation parameters for CS disintegration in the absence and presence of inhibitors as Eq. 6

6 $${\mathrm{ln}}{{\mathrm{I}}_{\mathrm{L}}}={\text{ lnA}} - {{\mathrm{E}}_{\mathrm{a}}}/{\mathrm{RT}}$$

**Fig. 4:**
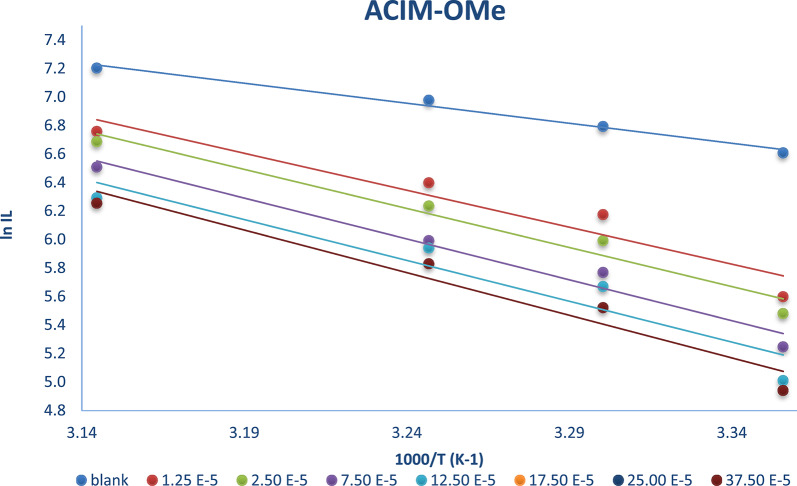
Arrhenius diagrams for calculating activation energy based on the relationship between temperature and limiting current with and without **ACIM-OMe**

Plotting *ln I*_*L*_ against *1000/T* as shown in Fig. 4 for **ACIM-OMe** and other **ACIMs** at Sup. Figures (23–25) yield straight lines; the values of *E*_*a*_ (energy of activation) are obtained from the slopes = *(-Ea/R*), where *A* is the factor of frequency and gas constant *R* = 8.314 JK^−1^mol^− 1^. Additionally, Table 5 contains the activation settings. Assuming that CS corrodes slowly in the presence of **ACIM** inhibitors, it is found that the unfettered solution’s *E*_*a*_ is lower than the inhibited solution’s. Analyzing the data reveals that *E*_*a*_ values rise when any inhibitor is present [36], and that **ACIM-NO**_**2**_ values are the lowest while **ACIM-OMe** values are the highest. This suggests that the inhibitor’s chemical makeup affects the energy barrier that separates the reactants and the activated complex. This data illustrates how the inhibitors can limit consumption due to the increased energy barrier for CS dissolution. The formation of a surface coating layer acts as a mass exchange border and an energy barrier, increasing the activation energy [21, 22].

The frequency factor (*A*) in the Arrhenius equation represents the number of effective molecular collisions per unit time that can lead to a successful reaction. It reflects not only the collision frequency but also the correct orientation of molecules required for the reaction to proceed. In the context of corrosion and inhibition, a higher *A* value suggests that the CS surface and the aggressive species (in this case, H_3_PO_4_) undergo more frequent effective collisions. However, when inhibitors (**ACIM** derivatives) are present, the values of *A* increase dramatically compared to the blank. This indicates that the inhibitors significantly alter the reaction pathway, requiring higher energy and more specific orientations for dissolution to occur.

The variation of *A* among the inhibitors (highest for **ACIM-OMe**, lowest for **ACIM-NO**_**2**_) suggests that the substituent groups on the **ACIM** molecules influence molecular alignment and adsorption strength. This reveals how the inhibitor’s chemical makeup controls the balance between collision frequency and successful adsorption [37, 38].

**Table 5:** The values of the activation parameters *E*_*a*_, *∆H*^*≠*^, *∆S*^*≠*^, and *∆G*^*≠*^ were determined in 8 M H_3_PO_4_, both in the absence and presence of **ACIM** at different concentrations

ILs	C x10^5^ mol/L	E_a_ kJ/ mol	A x 10^-6^	∆S^≠^ J/mol.K	∆H^≠^ kJ/mol	∆G^≠^ kJ/mol
ACIM-OMe	Blank	23.33	9.32	-93.60	20.80	57.31
	2.94	43.10	11209.19	-61.00	40.57	59.13
	5.88	45.54	25603.63	-54.14	43.01	59.49
	8.82	47.62	46420.17	-49.19	45.09	60.06
	14.7	47.59	39438.17	-50.54	45.06	60.44
	20.58	49.67	81592.24	-44.50	47.14	60.68
ACIM-H	Blank	23.33	9.32	-93.60	20.80	57.31
	2.94	37.30	1472.17	-77.88	34.77	58.47
	5.88	39.99	3860.26	-69.87	37.46	58.72
	8.82	40.58	4304.81	-68.96	38.05	59.03
	14.7	42.05	7306.28	-64.56	39.52	59.17
	20.58	42.93	10071.75	-61.89	40.40	59.23
ACIM-Cl	Blank	23.33	9.32	-93.60	20.80	57.31
	2.94	32.45	248.51	-92.67	29.92	58.12
	5.88	34.34	459.66	-87.56	31.81	58.45
	8.82	39.70	3154.20	-71.54	37.17	58.94
	14.7	40.04	3465.07	-70.76	37.51	59.04
	20.58	40.84	4635.45	-68.34	38.31	59.10
ACIM-NO_2_	Blank	23.33	9.32	-93.60	20.80	57.31
	2.94	31.47	182.45	-95.24	28.94	57.91
	5.88	34.62	556.40	-85.97	32.09	58.25
	8.82	33.96	387.41	-88.98	31.43	58.50
	14.7	35.13	594.36	-85.42	32.60	58.59
	20.58	34.45	445.18	-87.82	31.92	58.65

The substitutional recipe of the Arrhenius equation, change state condition, is commonly used to calculate the enthalpy *∆H*^*≠*^ and entropy *∆S*^*≠*^ of activation as Eq. 7:

7 $${\mathrm{ln}}\left( {{\mathrm{I}}_{{\mathrm{L}}} /{\mathrm{T}}} \right){\mkern 1mu} = {\mkern 1mu} {\mathrm{ln}}\left( {{\mathrm{R}}/{\mathrm{Nh}}} \right){\text{ }} + {\text{ }}(\Delta {\mathrm{S}}^{ \ne } /{\mathrm{R}}) - \left( {\Delta {\mathrm{H}}^{ \ne } /{\mathrm{RT}}} \right) $$

where *T* is the temperature, *N* is Avogadro’s number, and *h* is Planck’s constant. We were able to verify the established thermodynamic response between the *Ea* and *∆H*^*≠*^ as Eq. 8 since the *Ea* and *∆H*^*≠*^ values shifted similarly [38].

8 $$\Delta H^{ \ne } = E_{a} - RT $$

Positive values for *∆H*^*≠*^ indicate that the activated compound forms through an endothermic mechanism, A gaseous reaction that is part of the corrosive process is indicated by *E*_*a*_ values larger than *∆H*^*≠*^. It is possible to determine *∆S*^*≠*^ values from the intercepts (equivalent to *ln (R/Nh) + (∆S*^*≠*^*/R)*) by plotting *ln (I*_*L*_*(*_*ACIM*_*))/T)* vs. *1000/T*. The inhibitors’ negative S-values showed that the activated complex in the rate-determining phase deals with association rather than dissociation, indicating that ordering increases as one moves from reactants to the active complex [39]. Using the Arrhenius equation, the change in free energy activation *(∆G*^*≠*^*)* was calculated. 9 [40, 41]:

9 $$ \Delta {\mathrm{G}}^{ \ne } {\text{ }} = \Delta {\mathrm{H}}^{ \ne } - {\mathrm{T}}\Delta {\mathrm{S}}^{ \ne } $$

In the inhibited situation, *∆G*^*≠*^ values are positive and rise more than in the blank case. According to the activation parameter data, physical composition affects the inhibition.

This increase indicates that the corrosion process becomes less thermodynamically favorable upon the addition of the inhibitor. The elevated *∆G*^*≠*^ values reflect a higher energy barrier for the transformation from reactants to the activated complex, confirming that the inhibitors effectively reduce the spontaneity of the dissolution reaction [42]. These findings highlight the role of the inhibitors’ molecular structure and physical composition in enhancing resistance to corrosion by stabilizing the CS surface and increasing the free energy of activation [43].

### Weight loss methodology

One of the traditional techniques for assessing a corrosion inhibitor’s effectiveness is the weight loss methodology. This technique is among the most popular and straightforward approaches because of its ease of use and applicability. It can be used in conjunction with electrochemical techniques to help address these limitations. Using the weight loss technique, the corrosion inhibition efficiency (%I_Eff_) and θ of the investigated ionic liquid inhibitors have been calculated using Eqs. 10 and 11 [24, 44]:

10 $$\Delta {\mathrm{W}}\,=\,{{\mathrm{W}}_{\mathrm{1}}}-{\text{ }}{{\mathrm{W}}_{\mathrm{2}}}$$

11 $$\% {{\mathrm{I}}_{{\mathrm{Eff}}}}=\left( {1 - \frac{{\Delta {\mathrm{W}}{{\text{}}_{cpds}}}}{{\Delta {\mathrm{W}}{{\text{}}_{Blank}}}}} \right) \times {\mathrm{1}}00\,=\,\theta \,{\text{x 1}}00$$

where *W*_*1*_and *W*_*2*_ are the weights before and after corrosion, respectively, and *∆W* is the weight loss in the absence (blank) and presence of inhibitors. The inhibition efficiency has been recorded for different concentrations, times, and temperatures [45]. We found that as the concentration of inhibitors increased at the same optimum temperature of 298 K, the weight loss decreased, and the inhibition efficiency increased. Additionally, as time increased, efficiency increased due to the formation of an adherent protective layer on the CS surface, resulting from the adsorption of inhibitors. By the way, in the comparison between **ACIM-OMe** and **ACIM-NO**_**2**_, we found that **ACIM-OMe** has better inhibition than **ACIM-NO**_**2**_ due to the donating (methoxy) and drawing (nitro) groups, which affect the adsorption power of the inhibitors in Table 6. On the other hand, for the same inhibitor **ACIM-OMe**, as the temperature increased, the inhibition efficiency decreased due to the inhibitor desorbing from the CS surface [46], as a result of the weak connections being readily broken, as shown in Table 7.

**Table 6:** Data on weight loss at high and low concentrations for **ACIM-OMe** and **ACIM-NO**_**2**_ at various times at 298 K

ILs	C x 10^5^ (M)	0.5 h		1 h		2 h		4 h		24 h	
		∆ W	% I_Eff_	∆ W	% I_Eff_	∆ W	% I_Eff_	∆ W	% I_Eff_	∆ W	% I_Eff_
ACIM-OMe	Blank	0.0580	-	0.1090	-	0.2590	-	0.4730	-	0.7360	-
	2.94	0.0369	36.3793	0.0641	41.1927	0.1430	44.7876	0.2530	46.5116	0.3370	54.2120
	5.88	0.0303	47.7586	0.0515	52.7523	0.1150	55.5985	0.1860	60.6765	0.2490	66.1685
	8.82	0.0268	53.7931	0.0419	61.5596	0.0911	64.8263	0.1460	69.1332	0.1970	73.2337
	14.7	0.0201	65.3448	0.0318	70.8257	0.0724	72.0463	0.0920	80.5497	0.1360	81.5217
	20.58	0.0180	68.9655	0.0300	72.4771	0.0681	73.7066	0.0880	81.3953	0.1260	82.8804
ACIM-NO_2_	Blank	0.0580	-	0.1090	-	0.2590	-	0.4730	-	0.7360	-
	2.94	0.0483	16.7241	0.0853	21.7431	0.1980	23.5521	0.3520	25.5814	0.5390	26.7663
	5.88	0.0465	19.8276	0.0811	25.5963	0.1790	30.8880	0.3100	34.4609	0.4630	37.0924
	8.82	0.0436	24.8276	0.0765	29.8165	0.1730	33.2046	0.2950	37.6321	0.4310	41.4402
	14.7	0.0415	28.4483	0.0720	33.9450	0.1680	35.1351	0.2860	39.5349	0.4120	44.0217
	20.58	0.0389	32.9310	0.0698	35.9633	0.1540	40.5405	0.2630	44.3975	0.3930	46.6033

**Table 7:** Data on weight loss for high concentration at 298 K and 318 K for **ACIM-OMe** at different times

ILs	T	C x 10^5^ (M)	0.5 h		1 h		2 h		4 h		24 h	
			∆ W	% I_Eff_	∆ W	% I_Eff_	∆ W	% I_Eff_	∆ W	% I_Eff_	∆ W	% I_Eff_
ACIM-OMe	298 K	Blank	0.0580	-	0.1090	-	0.2590	-	0.4730	-	0.7360	-
		20.58	0.0180	68.9655	0.0300	72.4771	0.0681	73.7066	0.0880	81.3953	0.1260	82.8804
	318 K	Blank	0.1790	-	0.3520	-	0.6130	-	0.9850	-	1.3870	-
		20.58	0.1180	34.0782	0.2150	38.9205	0.3630	40.7830	0.5710	42.0305	0.6530	52.9200

On the other hand, the thickness loss of CS can be calculated from Eq. 12 [47]:

12 $$ D = \frac{{\Delta Wx10^{4} }}{{\rho xS}} $$

where *D* is the thickness loss (cm), *ρ* is the density of CS (7.85 g/cm^3^), and *S* is the surface area (2 cm x 2 cm) [48]. The data in Table 8 showed that as the concentration of inhibitors increased, the thickness loss of CS decreased, because a greater concentration of inhibitors promotes the creation of protective films, which lowers CS dissolution and, consequently, the gradual loss of CS thickness. In addition to Table 9, the increasing temperature caused the thickness loss to increase, due to the protective film breaking down and the inhibitors losing stability on the CS surface. The **ACIM-OMe** thickness loss is lower than that of **ACIM-NO**_**2**_, indicating that **ACIM-OMe** has a higher ability to inhibit corrosion compared to other inhibitors [49].

**Table 8:** Data on thickness loss at high and low concentrations for **ACIM-OMe** and **ACIM-NO**_**2**_ at different times for 298 K

ILs	C x 10^5^ (M)	0.5 h	1 h	2 h	4 h	24 h
		D	D	D	D	D
ACIM-OMe	Blank	18.4713	34.7134	82.4841	150.6369	234.3949
	2.94	11.7516	20.4140	45.5414	80.5732	107.3248
	5.88	9.6497	16.4013	36.6242	59.2357	79.2994
	8.82	8.5350	13.3439	29.0127	46.4968	62.7389
	14.7	6.4013	10.1274	23.0573	29.2994	43.3121
	20.58	5.7325	9.5541	21.6879	28.0255	40.1274
ACIM-NO_2_	Blank	18.4713	34.7134	82.4841	150.6369	234.3949
	2.94	15.3822	27.1656	63.0573	112.1019	171.6561
	5.88	14.8089	25.8280	57.0064	98.7261	147.4522
	8.82	13.8854	24.3631	55.0955	93.9490	137.2611
	14.7	13.2166	22.9299	53.5032	91.0828	131.2102
	20.58	12.3885	22.2293	49.0446	83.7580	125.1592

**Table 9:** Thickness loss data for high concentrations at 298 K and 318 K for **ACIM-OMe** at various times

ILs	T	C x 10^5^ (M)	0.5 h	1 h	2 h	4 h	24 h
			D	D	D	D	D
ACIM-OMe	298 K	Blank	18.4713	34.7134	82.4841	150.6369	234.3949
		20.58	5.7325	9.5541	21.6879	28.0255	40.1274
	318 K	Blank	57.0064	112.1019	195.2229	313.6943	441.7197
		20.58	37.5796	68.4713	115.6051	181.8471	207.9618

### Elemental speciation analysis

Many elements can be determined at trace and ultratrace levels using atomic absorption spectroscopy AAS, a reasonably selective detector with high sensitivity and spectrometric capabilities. Additionally, this analytical technique is a highly effective and potent tool for estimating corrosion rates in various environments, including acidic, neutral, and basic media, by assessing the solubility of corrosion byproducts.

Based on the amounts of iron (*Fe*^*+ 2*^) in the protected and unprotected systems, the absorbance percentage inhibition efficiency *(% €AAS*) of the ILs on the CS surface in 8M H_3_PO_4_ solution was computed using Eq. 13 [50, 51]. The AAS data in the Table 10 indicate that solutions containing **ACIM-OMe** inhibitor after corrosion have lower concentrations of *Fe*^*+ 2*^ for the optimum temperature and the highest concentration compared to solutions with or without inhibitors. This suggests that the **ACIM-OMe** inhibitor is more effective, has a promising indication, and a positive approach for inhibiting the corrosion of CS compared to others [52, 53].

On the other hand, the solution with or without the inhibitor was left for a week to indicate its efficiency over the long run. We found that the inhibition efficiency increased from 65.19% to 77.17% for the **ACIM-OMe** inhibitor. This suggests a more adsorption process of the inhibitor on the CS surface, which further suppressed the corrosion process.

13 $$\% {\mathrm{€}} {\text{AAS }}=\left( {{\mathrm{1}} - \frac{{{C_{Cpds}}}}{{{C_{blank}}}}} \right) \times 100$$

where *C*_*blank*_ and *C*_*ACIM*_ represent the concentrations of (*Fe*^*+ 2*^) ions, both without and with the inhibitors.

**Table 10:** Data from AAS demonstrate how **ACIM** affects iron ions at varying temperatures and concentrations, as well as the percentage inhibition efficiency of absorbance (% AAS)

Samples	[Fe^+2^] (mg.L^-1^)	Signal absorbance	%€AAS
Iron + 8 M H_3_PO_4_ (298 K)	70.00	0.14987	-
Iron + 8 M H_3_PO_4_ + 20.58 × 10^-5^ M ACIM-OMe (298 K)	24.37	0.05416	65.19
Iron + 8 M H_3_PO_4_ + 20.58 × 10^-5^ M ACIM-H (298 K)	33.94	0.07469	51.51
Iron + 8 M H_3_PO_4_ + 20.58 × 10^-5^ M ACIM-Cl (298 K)	36.25	0.07960	48.21
Iron + 8 M H_3_PO_4_ + 20.58 × 10^-5^ M ACIM-NO_2_ (298 K)	49.38	0.10729	29.46
Iron + 8 M H_3_PO_4_ (298 K) for a week	477.40	0.43030	-
Iron + 8 M H_3_PO_4_ + 20.58 × 10^-5^ M ACIM-OMe (298 K) for a week	109.00	0.27710	77.17

### SEM corrosion morphology and EDX analysis

Micrographs of the CS surface obtained via SEM under harsh acidic conditions were evaluated to corroborate the findings mentioned above. The various elements on the CS surface of each film can be identified using the EDX technique, along with the elemental changes that occur during immersion in an 8M H_3_PO_4_ solution, both in the absence and presence of different **ACIM** derivatives, at varying temperatures and concentrations, as depicted in the same Figure.

**Fig. 5:**
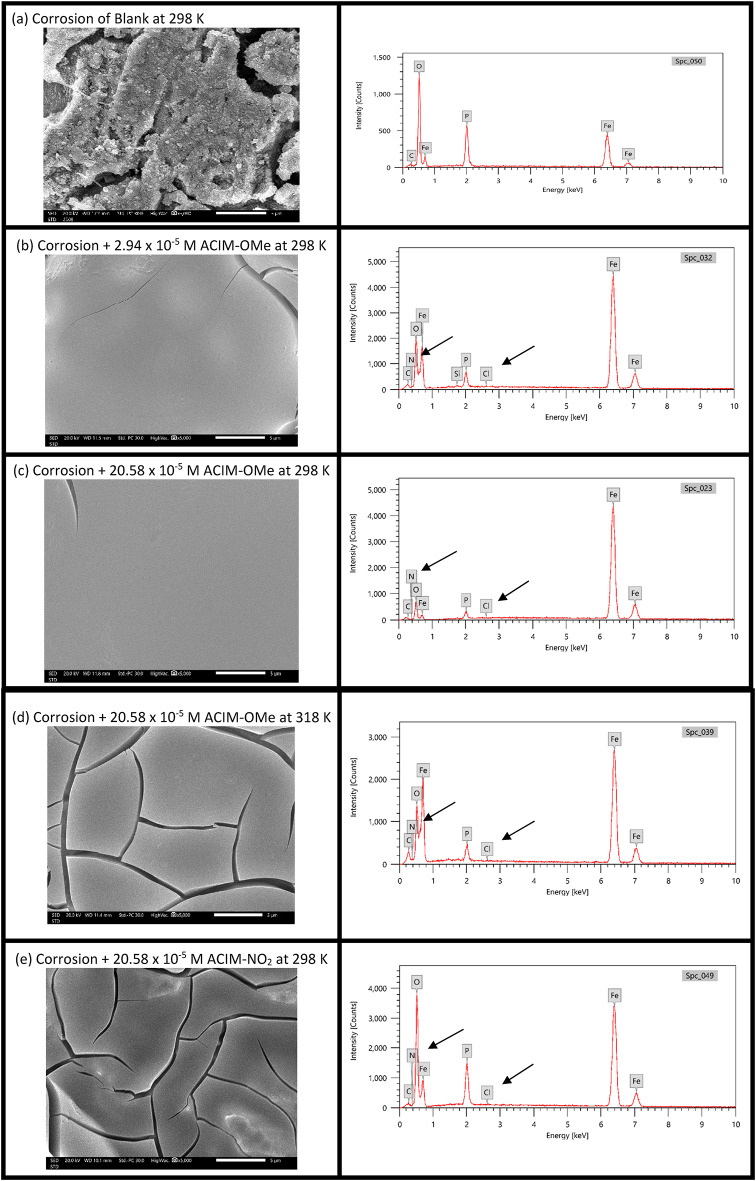
SEM and EDX of CS surface with and without **ACIM** at different temperatures and concentrations. **a** CS without **ACIM**, **b** CS with 2.94 × 10^− 5^M **ACIM-OMe** at 298 K, **c** CS with 20.58 × 10^− 5^ M **ACIM-OMe** at 298 K, **e** CS with 20.58 × 10^− 5^ M **ACIM-NO**_**2**_ at 298 K and **d** CS with 20.58 × 10^− 5^ **ACIM-OMe** at 318 K

SEM analysis at 5000× magnification (Fig. 5) revealed severe surface irregularities and pitting on the uninhibited CS sample due to aggressive attack by 8 M H₃PO₄. In contrast, samples treated with **ACIM** derivatives displayed significantly smoother morphologies, attributed to the adsorption of inhibitor molecules that form a protective barrier, thereby suppressing corrosion [54].

According to Fig. 5, the smoothness and inhibition efficiency varied with inhibitor concentration, temperature, and substituent type. At 298 K, higher concentrations of **ACIM-OMe** produced visibly smoother surfaces than lower concentrations. Increasing the temperature to 318 K at the highest concentration (20.58 × 10⁻⁵ M) reduced surface smoothness and inhibition efficiency, likely due to partial inhibitor desorption. Nevertheless, **ACIM-OMe** formed a denser and more uniform protective layer compared to **ACIM-NO₂** and other derivatives under identical conditions, correlating with its superior corrosion inhibition performance [55, 56].

To quantify the surface changes, EDX analysis was performed, with elemental compositions summarized in Table 11. For the blank sample (Fig. 5a), the surface composition was *Fe* = 37.32%, C = 1.21%, *O* = 45.81%, and *P* = 15.66%, indicating substantial oxidation and phosphate deposition due to corrosive attack. However, this composition is altered after the addition of **ACIM-OMe** (20.58 × 10⁻⁵ M, 298 K; Fig. 5c), the *Fe* and *C* content increased to 91.27%, and 2.88 respectivey, while *O* and *P* decreased to 3.57% and 2.20%, respectively. This corresponds to a 92.20% reduction in *O* and an 85.95% reduction in *P* relative to the blank, quantitatively demonstrating effective suppression of corrosion products The presence of nitrogen (*N*) and chlorine (*Cl*) in the EDX spectra of inhibited samples confirmed the adsorption of **ACIM** molecules on the CS surface. Among the inhibitors, **ACIM-OMe** exhibited the highest Fe recovery and the greatest reduction in *O* and *P*, consistent with its superior inhibition efficiency (81.08%). In comparison, **ACIM-NO₂** (Fig. 5e) showed a less pronounced protective effect, with *Fe* = 61.13%, *C* = 1.69, *O* = 28.27%, and *P* = 8.80%, reflecting its lower inhibition performance [57, 58].

These quantitative EDX data directly correlate with the visual smoothness observed in SEM images and provide measurable evidence of surface coverage and corrosion mitigation. The results confirm that **ACIM-OMe** forms the most effective protective film, minimizing oxidative damage and maintaining surface integrity under acidic conditions.

**Table 11:** EDX spectra of **ACIM** were used to calculate the percentage of elemental analysis content

NO	Samples	Fe	C	O	*P*
a	Blank (298 K)	37.32	1.21	45.81	15.66
b	Corrosion + ACIM-OMe 2.94 × 10^-5^ M (298 K)	78.00	2.66	14.89	3.85
c	Corrosion + ACIM-OMe 20.58 × 10^-5^ M (298 K)	91.27	2.88	3.57	2.20
c	Corrosion + ACIM-OMe 20.58 × 10^-5^ M (298 K)	91.27	2.88	3.57	2.20
d	Corrosion + ACIM-OMe 20.58 × 10^-5^ M (318 K)	71.23	2.38	21.60	4.62
c	Corrosion + ACIM-OMe 20.58 × 10^-5^ M (298 K)	91.27	2.88	3.57	2.20
e	Corrosion + ACIM-NO_2_ 20.58 × 10^-5^ M (298 K)	61.13	1.69	28.27	8.80

### X-ray photoelectron spectroscopy (XPS)

**Fig. 6:**
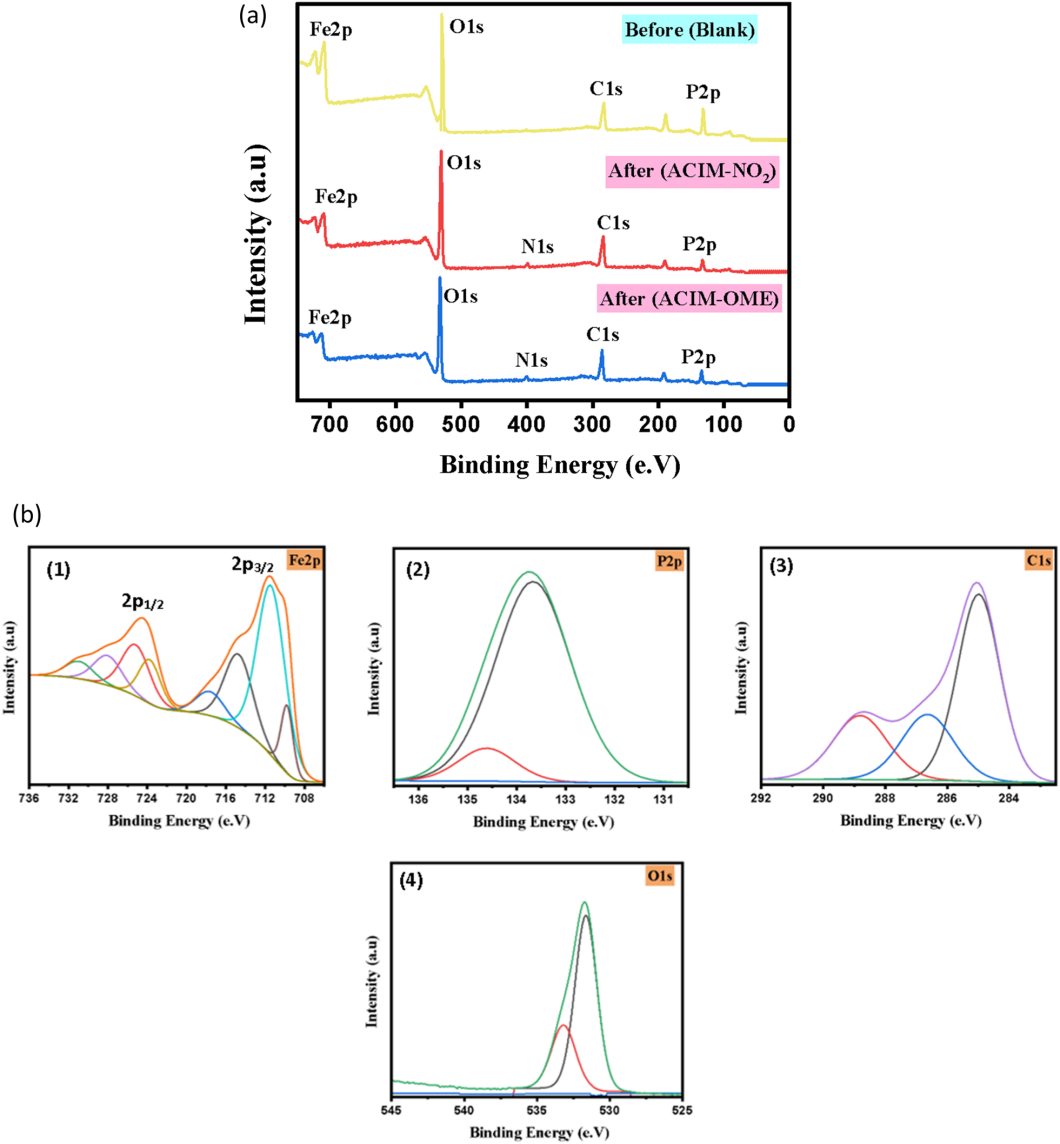

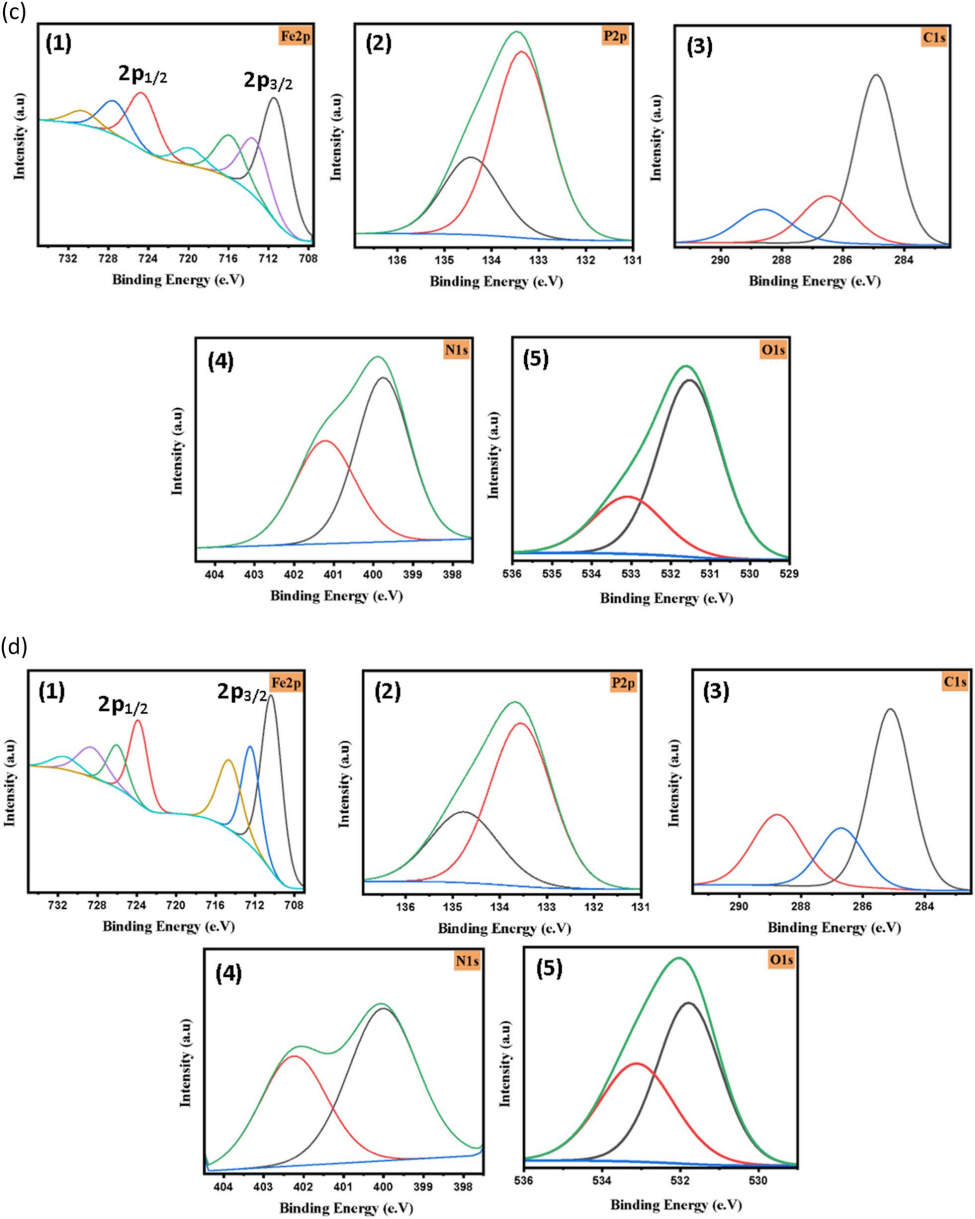
**a** XPS scan of Iron before and after adsorption, **b** XPS scan of Blank (without **ACIM)** (1) Fe2p, (2) P2p (3) C1s, (4) O1s, **c** XPS scan of **ACIM-OMe** after adsorption on CS (1) Fe2p, (2) P2p (3) C1s, (4) N1s, (5) O1s and **d** XPS scan of **ACIM-NO**_**2**_ after adsorption on CS (1) Fe2p, (2) P2p (3) C1s, (4) N1s, (5) O1s

X-ray photoelectron spectroscopy (XPS) was employed to gain deeper insight into the surface chemical composition, oxidation states of the constituent elements, and the nature of the interaction between the CS surface and the inhibitor molecules. The survey spectra (Fig. 6a) reveal the presence of *Fe2p*,* P2p*,* C1s*,* N1s*, and *O1s* peaks for the adsorbed layer formed on the exposed CS surface after immersion in 8 M H₃PO₄ solution, both in the absence and presence of ACIM derivatives under galvanostatic conditions, confirming the formation of a surface film [59, 60].

The XPS spectrum of the blank CS sample (Fig. 6b) shows characteristic *Fe2p* peaks with binding energies at 711.48 eV (Fe 2p3/2) and 724.48 eV (Fe 2p1/2), which can be attributed to the coexistence of Fe^2+^ and Fe^2+^ species corresponding to iron oxides such as *FeO/Fe*_*3*_*O*_*3*_ and *Fe*_*2*_*O*_*3*_. The binding energy range between 709 and 714 eV further supports the presence of *Fe–O* and *Fe–O–C* bonds, indicating partial surface oxidation of the steel in the acidic medium. The *P2p* spectrum exhibits two deconvoluted peaks at 133.69 and 134.66 eV, which are assigned to phosphate species (*PO*_*3*_^*3-*^ and *PO*_*4*_^*3-*^), suggesting the participation of phosphate ions from the acidic medium in the formation of the surface film. The *C1s* spectrum displays three peaks at 284.99, 286.64, and 288.81 eV, corresponding *to C–C/C = C*,* C–O*, and *O–C = O* bonds, respectively. Additionally, the *O1s* spectrum shows two main components at 531.64 and 533.18 eV, which are attributed to *Fe–O* and *O–C* bonds, confirming the presence of oxygen-containing species on the CS surface [2, 61, 62].

After adsorption of the **ACIM** derivatives, significant changes are observed in the XPS spectra. Notably, new *N1s* peaks appear, providing strong evidence for the adsorption of inhibitor molecules onto the CS surface through chemical interaction. As shown in Fig. 6c, the **ACIM-OMe** inhibitor exhibits two N1s peaks at 399.54 and 400.64 eV, which are attributed to *C–N* and *Fe–N* bonding, respectively. These features indicate the formation of a compact protective layer that hinders the corrosion process.

Moreover, slight shifts of *Fe*,* N*,* P*, and *O* peaks toward lower binding energies after inhibitor adsorption suggest electron donation from the inhibitor molecules to the CSsurface, leading to stronger interfacial interactions and enhanced surface stability. Similar behavior is observed for the **ACIM-NO**_**2**_ inhibitor (Fig. 6d), where the appearance of *N1s* peaks and the systematic binding energy shifts (summarized in Table 12) confirm the formation of an adsorbed protective film [20, 63]. Overall, the XPS results demonstrate that **ACIM** derivatives adsorb on the CS surface through a mixed physisorption–chemisorption mechanism, involving electrostatic interactions as well as coordination between nitrogen-containing functional groups and iron atoms, which explains their effective corrosion inhibition performance.

**Table 12:** XPS binding energies for **ACIM** after adsorption

ILs	Chemical species	B.E (eV)	ILs	Chemical species	B.E (eV)
ACIM-OMe	Fe 2p3/2	711.09	ACIM-NO_2_	Fe 2p3/2	709.74
	Fe 2p1/2	724.57		Fe 2p1/2	723.51
	P2p	133.76		P2p	133.94
		133.61			133.89
	C1s	284.91		C1s	285.1
		286.5			288.77
		288.59			286.7
	N1s	399.54		N1s	399.77
		400.64			401.67
	O1s	531.52		O1s	531.76
		533.08			532.74

### Antibacterial activity

**Fig. 7:**
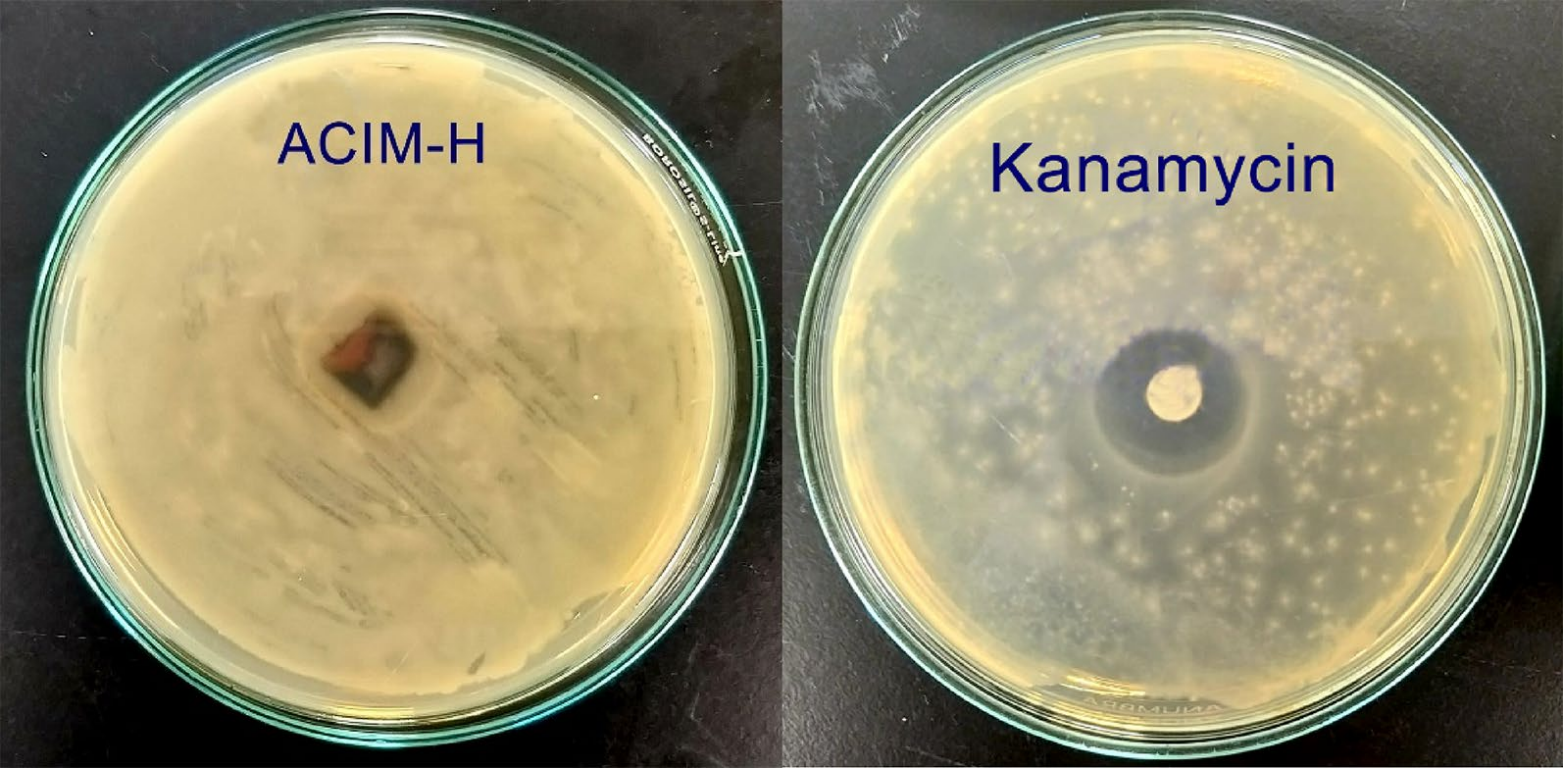
Antibacterial activity comparison between **ACIM-H** and Kanamycin antibiotic

The tested inhibitors were applied to the disk of the CS plate. The inhibition zone was calculated by measuring the diameter of the zone around the CS disk (mm), including the disk diameter, and comparing it with 10 µl/1 mL of the standard antibiotic Kanamycin. The readings were taken in three different fixed directions in all triplicates, and the average values are presented in Table 13. Antibacterial testing revealed that only **ACIM-H** exhibited measurable antibacterial activity against Salmonella spp., while **ACIM-OMe**,** ACIM-Cl**, and **ACIM-NO₂** showed no detectable inhibition zones under the tested conditions, as shown in Fig. 7 [64, 65].

**Table 13:** Antibacterial activity for the inhibition zone of **ACIM**

Bacterial Sample	Salmonella G -
	Inhibition Zone in millimeters (mm)
ACIM-OMe	-ve
ACIM-H	17.50
ACIM-Cl	-ve
ACIM- NO_2_	-ve
Kanamycin antibiotic	29.50

### Theoretical computational investigation study

**Fig. 8:**
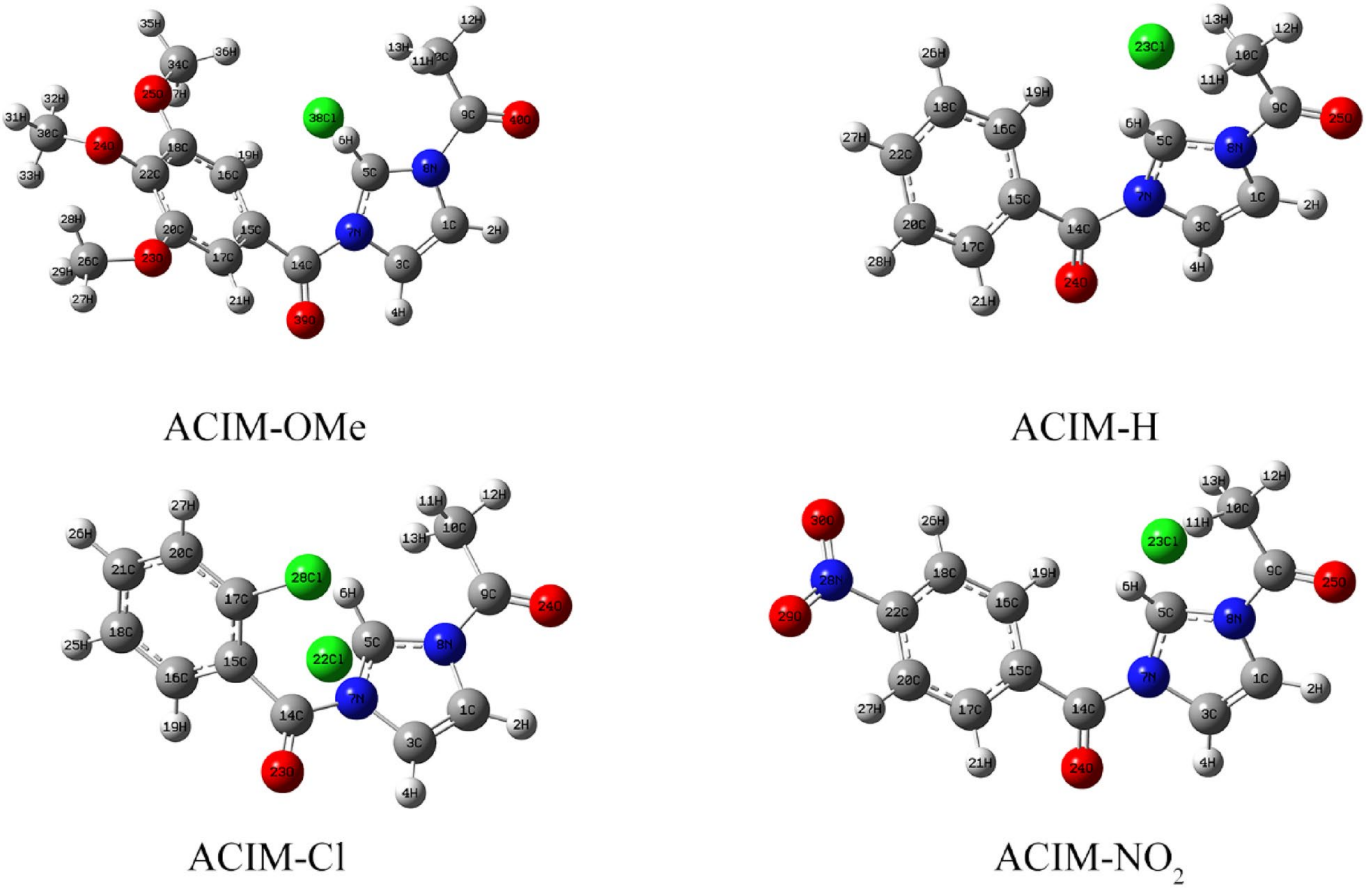
Optimized geometric configuration of **ACIM** structure

*E*_*HOM*O_, *E*_*LUMO*_, energy gap (*∆E = E*_*LUMO*_ *- E*_*HOMO*_), chemical hardness, softness, electronegativity, chemical potential, proton affinity, electrophilicity, and nucleophilicity are examples of quantum chemical descriptors that have been proven to be highly successful and helpful in CS corrosion investigations. Table 14 provides a summary of the global molecular characteristics and properties of the chosen inhibitors in both the gas phase and aqueous solution [66], as well as the geometry optimization structures shown in Fig. 8. The impact of each descriptor on the order of inhibition efficiencies will be covered in the sections that follow. The frontier molecular orbital (FMO) concept is related to a particle’s ability to adsorb onto the CS surface. *E*_*HOMO*_ alludes to the capacity of atoms to donate electrons to the metallic iron surface, which possesses empty “*d*” orbitals. The ligand is more likely to give electrons to the iron atoms to create a stronger binding when the *HOMO* energy level (*E*_*HOMO*_) is higher. This could demonstrate how well the substance inhibits the ability of atoms to donate electrons. Based on this, we conclude that **ACIM-OMe** is better equipped to provide electrons than other inhibitory particles in the two phases. The *E*_*HOMO*_ of **ACIM-OMe** is the largest, which is consistent with the practical results. As a result, this compound is thought to be more suitable for adsorption on the metallic surface via the pyridine ring, which is a rich source of electrons. However, *LUMO* energy shows how the atoms secure the electrons. The effectiveness of the inhibitor is more noticeable when the *E*_*LUMO*_ is lower. A vital boundary for the inclination of particle reactivity towards the CS surface is the energy gap (*∆E*_*gap*_). The likelihood that atoms will adsorb on the CS surface increases with decreasing *∆E*_*gap*_. The results show that *∆E*_*gap*_ values are generally smaller in the aqueous phase than in the gas phase, confirming that adsorption is more favorable in solution [66]. In contrast, **ACIM-NO**_**2**_, which contains a strong electron-withdrawing group, exhibits reduced electron density at its adsorption centers, higher ΔE values, and weaker adsorption capability, which explains its lower inhibition efficiency.

Notably, **ACIM-OMe** presented the lowest *∆E*_*gap*_ values in both phases, supporting its strong reactivity and inhibition efficiency. Density functional theory (DFT) was used to execute their FMOs at the *B3LYP/6-311* g (d, p) level of hypothesis, and to display the structural form in Fig. 8, which shows the *FMOs* for the concentrated-on inhibitors. The equations used to compute the additional parameters, such as electronegativity (*X*), work function (*∆N*), electron back donation (*∆E*_*b.d*_), hardness (ƞ), and softness (*σ*), are described in Sup. Table 1. Where (*X*_*Fe*_) is the electronegativity of *Fe* = 7 eV, ionization potential (*I*), chemical potential( *µ)*, electron affinity (*A*), and hardness of *Fe* is (η_*Fe*_) = 0. The positive *∆N* values confirm electron transfer from the inhibitor molecules to the *Fe* surface. Moreover, *∆E*_*steel/inh*_ supports the strong interaction between the inhibitors and the carbon steel surface [19, 67].

Due to its high work function, high softness, low hardness, high electronegativity, and a lower negative value of back donation, the inhibitor **ACIM-OMe** inhibits more effectively than the others in both phases. While *ω*^*+*^, *ω*^*−*^, and *∆ω*^*±*^, according to four distinct principles, indicate electron accepting, electron donating, and net electrophilicity, respectively, electrophilicity (*ω*) denotes the acceptance of electrons for the molecule. These properties were found to increase as the energy gap decreased, thereby enhancing inhibition performance. Conversely, dipole moment (*µ*_*d*_) quantifies the polarity of the chemical bond in molecules with a bigger *µ*_*d*_ value of **ACIM-OMe** than others; a higher value of *µ*_*d*_ would indicate a more anticorrosive molecule, which is consistent with experimental findings [2, 54].

**Table 14:** Calculated quantum parameters for **ACIM** in gaseous and aqueous phases

State	Gas				Aqueous			
ILs	ACIM-OMe	ACIM-H	ACIM-Cl	ACIM-NO_2_	ACIM-OMe	ACIM-H	ACIM-Cl	ACIM-NO_2_
E_HOMO_ (eV)	-6.4172	-6.5762	-6.4725	-6.8621	-6.5756	-6.8273	-6.9032	-6.8777
E_LUMO_ (eV)	-2.5609	-2.3111	-1.9698	-0.5586	-2.9043	-2.9432	-3.0645	-0.7489
∆E_gap_ (eV)	3.8564	4.2651	4.5027	6.3035	3.6713	3.8841	3.8387	6.1288
I (eV)	6.4172	6.5762	6.4725	6.8621	6.5756	6.8273	6.9032	6.8777
A (eV)	2.5609	2.3111	1.9698	0.5586	2.9043	2.9432	3.0645	0.7489
X (eV)	4.4890	4.4436	4.2212	3.7104	4.7399	4.8852	4.9839	3.8133
μ (eV)	-4.4890	-4.4436	-4.2212	-3.7104	-4.7399	-4.8852	-4.9839	-3.8133
µ_d_ (Debye)	5.0183	4.5080	3.8339	3.5227	6.7927	6.1122	5.0291	4.1470
ƞ (eV)	1.9282	2.1325	2.2513	3.1517	1.8357	1.9421	1.9193	3.0644
σ (eV^-1^)	0.5186	0.4689	0.4442	0.3173	0.5448	0.5149	0.5210	0.3263
ω (eV)	5.2255	4.6296	3.9573	2.1840	6.1195	6.1444	6.4707	2.3726
∆E_b.d_ (eV)	-0.4820	-0.5331	-0.5628	-0.7879	-0.4589	-0.4855	-0.4798	-0.7661
∆N (eV)	0.6511	0.5994	0.6172	0.5219	0.6156	0.5445	0.5252	0.5200
T.E (Hartee)	-1527.9175	-1184.2682	-1643.8853	-1388.8202	-1527.9503	-1184.3007	-1643.9147	-1388.8543
∆E_steel/inh_ (eV)	0.8175	0.7661	0.8575	0.8584	0.6956	0.5757	0.5294	0.8285
ω^-^ (eV)	7.7111	7.1180	6.3492	4.4332	8.7190	8.8298	9.2026	4.6622
ω^+^ (eV)	3.2220	2.6743	2.1281	0.7228	3.9790	3.9445	4.2187	0.8490
∆ω^±^ (eV)	3.0923	2.5339	1.9706	0.4972	3.8643	3.8313	4.1100	0.6345

#### Topological analysis

**Fig. 9:**
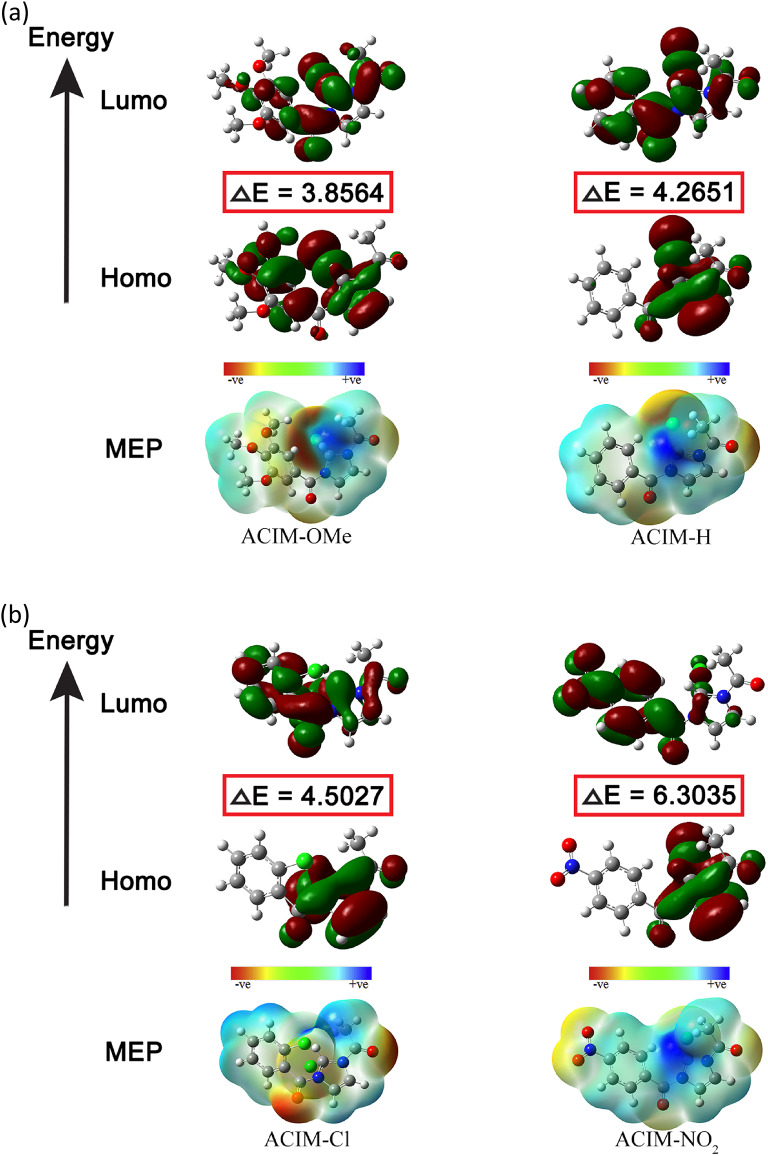

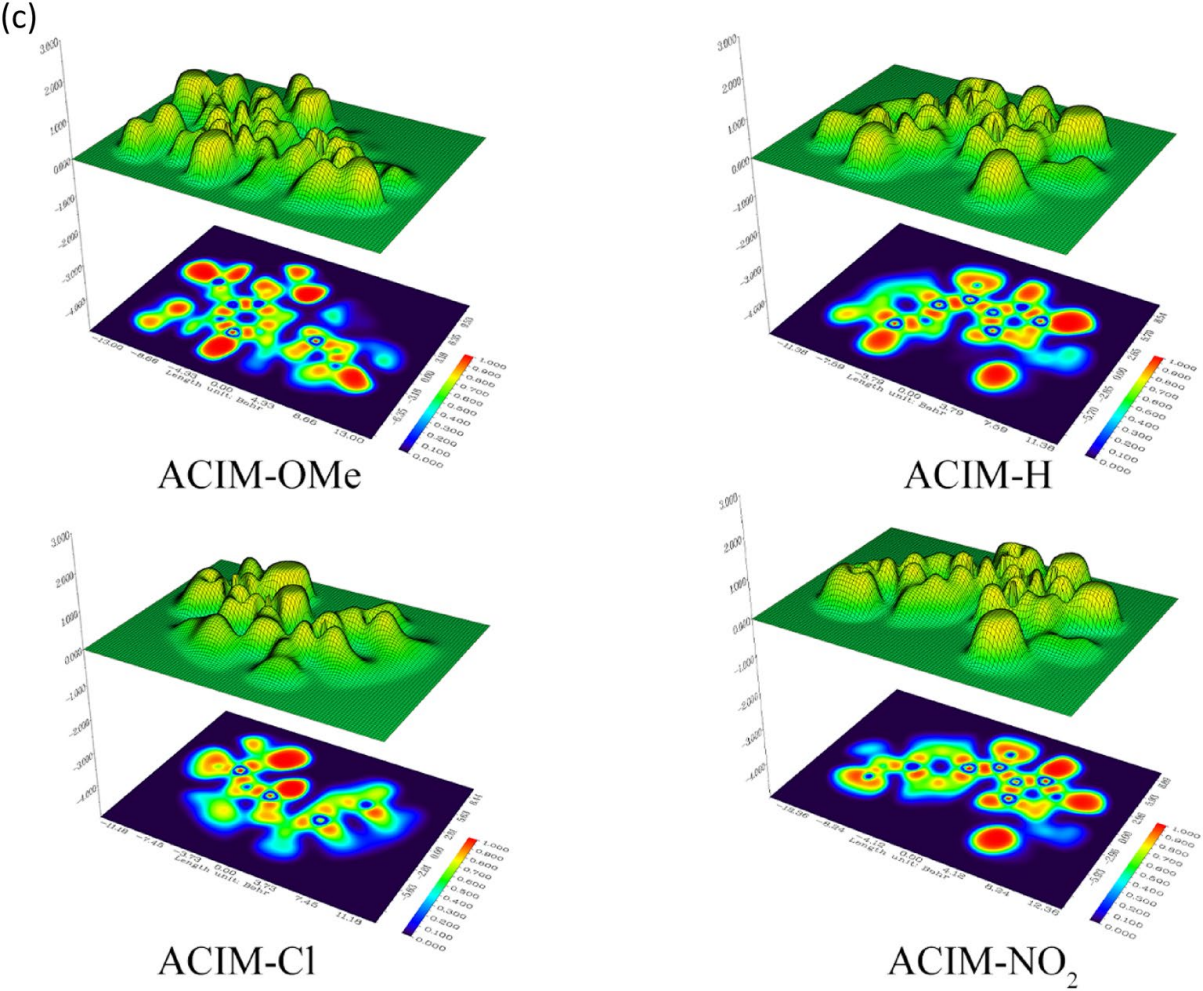
Frontier molecular orbital analysis for the energy distribution of HOMO-LUMO and molecular electrostatic potential **a ACIM-OMe** and **ACIM- H**, **b ACIM-Cl** and **ACIM-NO**_**2**_ and **c** Electron localization function for **ACIM** along XY plan

Electron localization zones on the surface of free and bound structures can be identified and predicted using topological analysis, including the electron localization function (*ELF*) [68] and molecular electrostatic potential (MEP) features, which demonstrate how the CS surface absorbs inhibitors. The *O* and *N* atoms for inhibitor matrices that carry negative charges are shown in Fig. 9(a-b) for MEP. The CS’s positive sites can exchange electrons with the significantly negatively charged atoms. Alternatively, Sup. Tables (2–3) displays the distribution of Mulliken’s atomic charges on **ACIM-OMe’s** atoms, particularly since **ACIM-OMe** has the highest inhibitory efficiency in anticorrosive processes. *N7*,* N8*,* O23*,* O24*,* O25*,* O39*,* O40* for **ACIM-OMe** are the best atoms in gas and aqueous phases. Therefore, a nucleophilic attack on the CS’s surface is caused by those atoms. The MEP and electron density (*ED*) zones are displayed in Figs. 9(a-b). Red indicates a high *ED*, whereas blue indicates a low *ED*. Red > orange > yellow > green > blue is the order in which *ED* declines. For inhibitors, the high *ED* (yellow to red color) is found on the *O* and *N* atoms. Other atoms have a low *ED*, which is green to blue in color [69]. The *ELF* surface and the color-filled map (along with the *XY* plan), on the other hand, offer helpful insights into donor interactions, as seen in Fig. 9c. The core of the map explains the highly electron localization (red color) around coordinated atoms (*N7*,* N8*,* O24*) for all inhibitors to succinct electron transfer, while the other atoms lower electron localization value (blue color) through all surface map planes. It is found on other atoms and has a green to blue hue [70].

#### Fukui indices and local dual descriptors

Fukui indices and local dual descriptors describe the electrophilic and nucleophilic attack in the gas phase. We used this study for the best inhibitor, Sup. Table 2 for **ACIM-OMe**. Displays the condensed Fukui functions derived using Mulliken charges (*f*_*k*_*+*, *f*_*k*_*-*), local electrophilicity (*ω*_*k*_*+*, *ω*_*k*_*-*), and local softness (*σ*_*k*_*+*, *σ*_*k*_*-*). We have computed (*Δf*_*k*_, *∆σ*, and *∆ω*) to make it easier to compare the potential locations for nucleophilic and electrophilic assaults on every atom *k*. This represents the difference between (*f*_*k*_*+*,* f*_*k*_*-*), (*σ*_*k*_*+*,* σ*_*k*_*-*), and (*ω*_*k*_*+*,* ω*_*k*_*-*), respectively [71].

The equations presented in Sup. Table 4 show that, when comparing atomic sites to identify the most reactive centers, positive values of *∆f*,* ∆σ*, and *∆ω* (> 0) indicate sites that are more favorable for nucleophilic attack, such as O23, O39, and O40. Conversely, negative values of these descriptors suggest sites more susceptible to electrophilic attack at other atomic positions.

So, we concluded that the experimental results show that the inhibition efficiency follows the order: **ACIM-OMe > ACIM-H > ACIM-Cl > ACIM-NO**_**2**_ .This trend is fully supported by the theoretical calculations and substituent electronic effects.

#### Molecular dynamics simulations (MD)

**Fig. 10:**
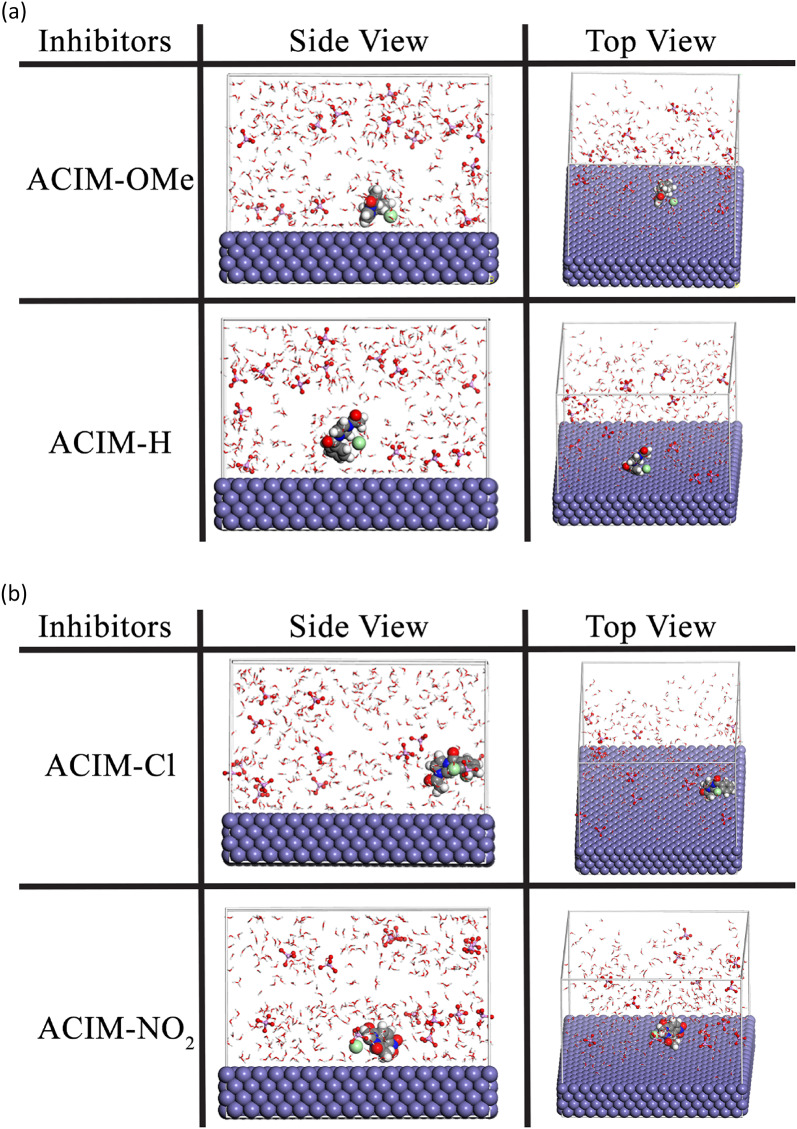
The optimized structure adsorption **a ACIM-OMe** and **ACIM- H**, **b ACIM-Cl and ACIM-NO**_**2**_

One essential theoretical method for MD simulating the adsorption process between the corrosion inhibitors and the surface of the CS substrate is molecular dynamics. Cleaving pure iron along the (110) plane was the first step in the modeling procedure [72]. Two layers were then integrated to create a simulation box. Layers of solvents that include either inhibitor, H_2_O molecules, or H_3_PO_4_ molecules. To investigate the known interactions between inhibitor molecules and CS surfaces, such as *Fe* (110), molecular dynamics (MD) simulations offer a useful theoretical method. The top and side views of the inhibitors’ optimal adsorption geometries on the *Fe* (110) surface are displayed in Figs. 10a-b. These compounds have polar functional groups and concentrated heteroatoms (nitrogen and oxygen) that allow for strong interactions with the vacant 3d atomic orbitals of iron. This type of molecule may provide complete surface coverage under ideal adsorption conditions, thereby bringing it into proximity to *Fe* atoms. Inhibitor molecules enhance contact with the iron surface by essentially aligning themselves parallel to it. Because of this, a protective barrier spreads out across the CS surface, keeping dangerous materials like H_3_PO_4_ molecules from soaking into the metal. This efficiently reduces corrosion [73].

### The role of Hammett and Taft constants in inhibitor coordination bonding

**Fig. 11:**
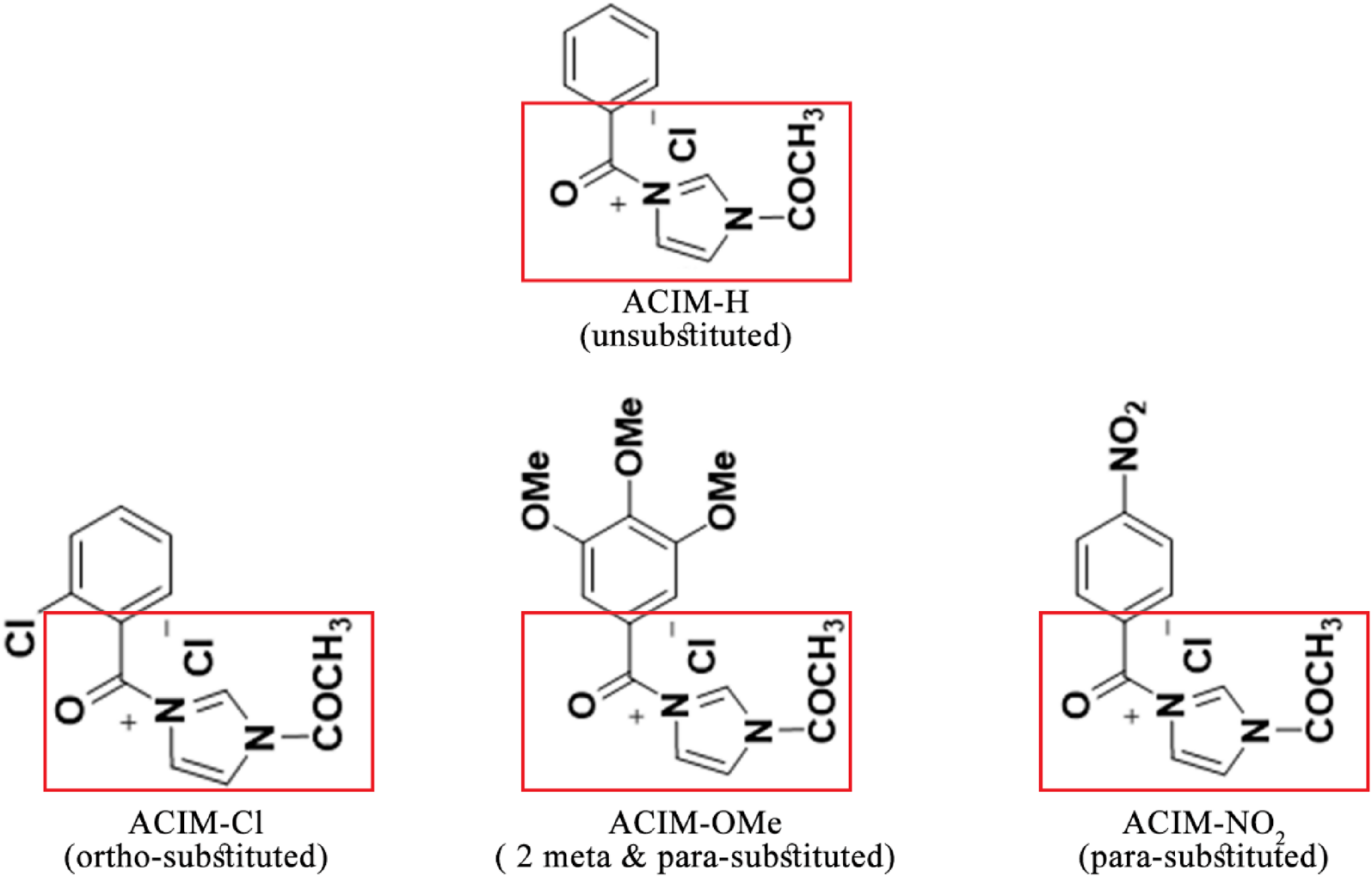
The relative electronic and steric effects of substituents **ACIM**

In organic chemistry, the Hammett equation is a helpful tool for explaining how substituents’ electronic effects affect the pace and equilibrium of organic processes. The Hammett equation has also been used extensively to study and characterize the interaction between an organic inhibitor, such as ionic liquid inhibitors, and a metallic substrate, such as the surface of CS. This also discusses how different substituents in organic compounds might inhibit corrosion. The Hammett equation is presented in Eqs. (1, 2) [74]. The type of reaction mostly determines its value. The ***σ*** parameter represents the electronic properties of an aromatic substituent. A substituent that donates electrons will have a negative ***σ*** value, whereas one that withdraws electrons will have a positive ***σ*** value. By calculating $\mathrm{log}((1-{\%}{{IE}}_{R})/(1-{\%}{{IE}}_{H}))$ vs. values of Hammett constants for -meta and -para substituents, the average value of (ρ) is negative (-0.3131), indicating stronger binding with electron-donating groups [75]. For **ACIM-OMe**, which contains three methoxy substituents, the total *Hammett* substituent constant (*σ*_*total*_) for a molecule with multiple substituents is typically calculated by summing the individual σ values of each substituent. On the other hand, the Hammett equation can’t be applied to -ortho substituents like -ortho chloro substituent imidazole derivative, because -ortho substituents are in proximity to the reaction centre (e.g., a carboxyl or electrophilic group), potentially inducing steric hindrance that influences reactivity, a factor not considered by Hammett *σ* values. The Taft dual-parameter equation is an extension of the Hammett equation [76], which considers both the electronic and steric effects of substituents, as shown in Fig. 11. According to a careful review of literature findings, the %I_Eff_ of organic ionic liquid inhibitors increases with the substituents’ capacity to donate electrons. They found that the following pattern is followed by the *%I*_*Eff*_ of ***-OMe***, ***-Cl***, and *-****NO***_***2***_ substituted imidazole derivatives: -***NO***_***2***_ ***< -Cl < -H < -OMe*** [75]. According to the results of this research, the presence of the ***-OMe*** group improves their %I_*Eff*_, which suggests that it has a good impact on their performance. However, the e-withdrawing ***-NO***_***2***_ substituent has a detrimental effect since it lowers their *%IE*. By creating a corrosion barrier, these materials facilitate their adsorption.

In particular, molecular dynamics (MD) simulations provide direct insight into the adsorption behavior of the inhibitors on the carbon steel surface by explicitly modeling the dynamic interaction between inhibitor molecules and the Fe (110) surface in a corrosive environment [77]. The calculated adsorption energy (E_ads_) serves as a quantitative measure of the interaction strength and adsorption stability, where more negative E_ads_ values indicate stronger, spontaneous adsorption and the formation of a more stable protective layer. As summarized in Eq. (14) [20], the calculated adsorption energies follow the order: **ACIM-OMe** (− 1075.10 kcal/mol) > **ACIM-H** (− 987.89 kcal/mol) > **ACIM-Cl** (− 914.68 kcal/mol) > **ACIM-NO**_**2**_ (− 826.62 kcal/mol). This theoretical trend is in excellent agreement with the experimentally observed inhibition efficiencies, confirming that ACIM-OMe, which exhibits the most negative Eads value, demonstrates the strongest adsorption affinity and the highest corrosion inhibition efficiency among the studied inhibitors.

14 $${{\mathrm{E}}_{{\mathrm{ads}}}}={\text{ }}{{\mathrm{E}}_{{\mathrm{Inhibitor}}/{\text{Metal ion}}}}-{\text{ }}\left( {{{\mathrm{E}}_{{\mathrm{Inhibitor}}}}+{\text{ }}{{\mathrm{E}}_{{\text{Metal ion}}}}} \right)$$

### Mechanistic insights into corrosion inhibition

**Fig. 12:**
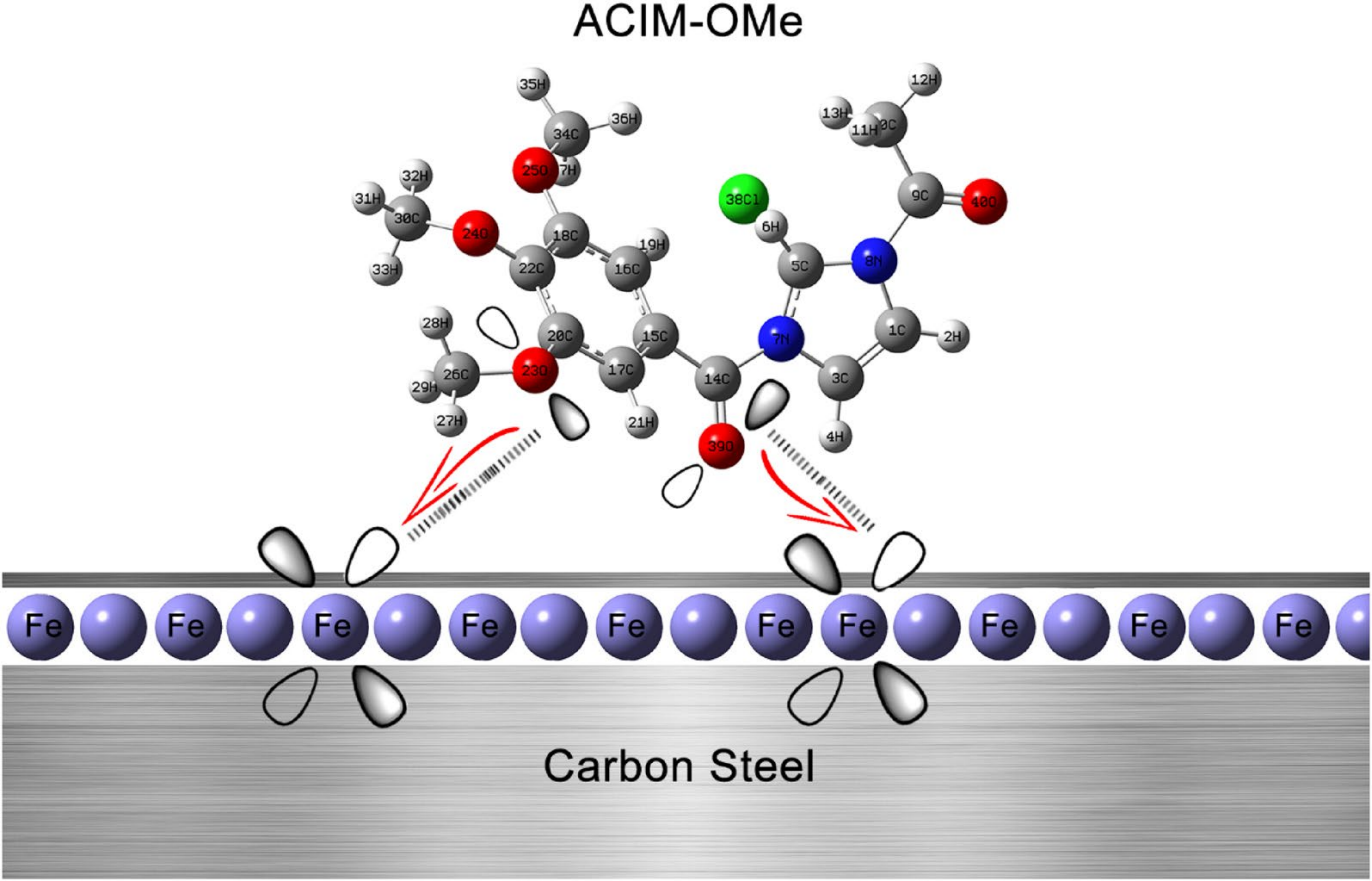
Diagrammatic mechanism of adsorption **ACIM-OMe** on the surface of CS in 8 M H_3_PO_4_

Based on the earlier discussion and the calculated values of free energy of adsorption, the corrosion inhibition of CS by ACIM under acidic conditions is likely governed by a mixed physisorption–chemisorption mechanism. The physisorption component may arise from the presence of charged species on the CS surface or from the protonation of the inhibitors’ heteroatoms in the bulk solution. As illustrated in Fig. 12 for **ACIM-OMe**, the chemisorption could be attributed to the charge transfer from the inhibitor’s electron-rich centers (the lone pair of the heteroatom or the π-electrons of the unsaturated centers) to the CS vacant d-orbitals (*Fe*^*2+*^) [78], which would reduce surface erosion. Since the order of polar groups in electron withdrawing is *OMe* > *H* > *Cl* > *NO*_*2*_, the presence of the **OMe** may reduce the amount of charge transfer from the inhibitor to the CS surface. The calculated Mulliken atomic charges of *O23* (-0.366394) & *O39* (-0.301196) of **ACIM-OMe** are more negative than those of other atoms for other inhibitors. These results highlight the superior inhibitory efficiency of **ACIM-OMe** compared to the other compounds [79, 80].

### Imidazole derivatives: comparing novel inhibitors with known analogue

The functionalized imidazole ionic liquids **ACIM** exhibit higher inhibition efficiency, similar to other analogs, by varying optimal concentrations and at the ideal temperature of 298 K for CS in different aggressive media, such as HCl or H_2_SO_4_, as shown in Table 15. Here we use H_3_PO_4,_ which can help to increase the efficiency and reduce the damage to the CS.

Unlike sulfuric and hydrochloric acids, phosphoric acid is relatively mild, yet its concentrated solutions (15%–70%) efficiently dissolve oxides. At lower concentrations, it forms a thin layer of iron phosphate, resulting in minimal corrosion. Widely used in the phosphate sector, phosphoric acid plays a vital role in fertilizer production, as well as in pharmaceutical and food applications [81].

The presence of two nitrogen atoms in the imidazole ring accounts for its enhanced performance, as these structural features promote adsorption and protective film formation on the carbon steel surface. Additionally, specific substituents further enhance the inhibitors’ protective capacity, resulting in greater corrosion resistance. Overall, these findings emphasize the significant advantages of the studied imidazole derivatives in mitigating corrosion and highlight their potential for advancing corrosion prevention strategies.

**Table 15:** Comparison between imidazole derivatives with different concentrations and media

ILs	Concentration(M)	%${I}_{Eff}$	Reference
	1 × 10^− 3^	87.6	[82]
	3 × 10^− 3^	95.8	[46]
	8 × 10^− 3^	90.6 93.3 92.9	[83]
	20.58 × 10^− 5^	81.08	This work

## Conclusion

The corrosion-inhibiting properties of ionic liquids containing imidazolium derivatives, specifically **ACIM-OMe**,** ACIM-H**,** ACIM-Cl**, and **ACIM-NO**_**2**_, were investigated in this research. Using spectroscopic methods, surface analysis, electrochemical trials, and theoretical insights, the investigation demonstrated that imidazolium-functionalized ionic liquids exhibited high inhibition efficiency at high concentrations at the optimal temperature of 298 K. The best inhibitor, **ACIM-OMe**, achieved an efficiency of 81.08%, and weight loss measurements validated these results. According to ∆*G*_ads_ data from the *El-Awady* model, the inhibitors adsorb onto the carbon steel surface through a physicochemical mechanism, forming a stable adsorption layer. Additionally, it was confirmed by characterizations employing SEM, EDX, XPS, and AAS techniques that the inhibitors of ionic liquids adhered to the surface. Antibacterial evaluation indicated that only **ACIM-H** displayed measurable activity against Salmonella spp., suggesting selective bioactivity among the series. While this property is ancillary to the primary corrosion inhibition mechanism, it may be relevant in environments where microbial corrosion is a concern and so supports broader application profiling.The Hammett equation correlates the electronic effects of substituents with the efficacy of corrosion inhibition. Laboratory results are in good agreement with DFT and MD models. Where Effective inhibitory potential was indicated by the DFT results, which showed that the investigated ILs have substantial adsorption and electron-donating capabilities towards the CS surface, MD simulations revealed that the protonated form adsorbed in a twisted manner, whereas the neutral form adsorbed flat on the iron surface.

Future studies should evaluate the long-term stability and inhibition performance of **ACIM** derivatives in real industrial effluents, saline, and elevated-temperature conditions to assess practical applicability. Expand antibacterial assessment to include broader microbial strains (e.g., sulfate-reducing bacteria) and biofilm formation tests to better evaluate potential in mitigating microbiologically influenced corrosion (MIC). Conduct comprehensive environmental impact assessments, including toxicity and biodegradability tests, to ensure the green credentials of these ILs. Explore the incorporation of **ACIM** compounds into protective coatings, paints, or polymeric matrices for extended surface protection in industrial applications.

## Supplementary Information

Below is the link to the electronic supplementary material.

Supplementary Material 1.

## Data Availability

The data used and analyzed during the current study are available from the corresponding authors upon reasonable request.
