# Supplementary material for: A multi-faceted study combining quantum chemical, Hammett parameters, and antibacterial assessment for imidazole: based green ionic liquids corrosion inhibition
Source: BMC Chem. 2026 Apr 14;20(1):89. doi: 10.1186/s13065-026-01775-4 (PMC13085551; doi:10.1186/s13065-026-01775-4)
Supplement: Supplementary file 1 — Supplementary Material 1. [file 13065_2026_1775_MOESM1_ESM.docx]

**A Multi-Faceted Study Combining Quantum Chemical, Hammett Parameters , and Antibacterial assessment for Imidazole - Based Green Ionic Liquids Corrosion Inhibition**

*Amira H. E. Moustafa , Hanaa H. Abdel-Rahman , Mohamed Hagar , Sherif A. A. Bishr*

Chemistry Department, Faculty of Science, Alexandria University, P.O. 426 Ibrahemia,

Alexandria 21321, Egypt.

**Characterization of imidazolium-based ionic liquids 3a-d**

- ***Characterization of 1-acetyl-3-benzoyl-1H-imidazol-3-ium chloride (ACIM-H)***

Colorless oil, m.p. = 15-17 °C, yield 94%. IR (*υ*, cm^-1^): 3075 (C-H ar), 2930 (C-H al), 1710 (C=O), 1620 (C=N), 1550 (C=C). ^1^H NMR (400 MHz, DMSO-*d_6_*): δ_H_ = 8.35-8.31 (2H, m, Ar-**H**), 8.14-8.11 (2H, m, Ar-**H**), 8.00 (1H, d, *J* = 12 Hz, Ar-**H**), 7.81 (1H, d, *J* = 12 Hz, Ar-**H**), 7.49-7.30 (2H, m, Ar-**H**), 2.52 (3H, s, C**H**_3_ overlapped with DMSO-*d_6_*). Molecular formula is C_12_O_2_N_2_H_11_Cl with molecular weight = 250.5 g/ mol, In Mass spectrometry = 249.08572 m/z.

- ***Characterization of 1-acetyl-3-(2-chlorobenzoyl)-1H-imidazol-3-ium chloride (ACIM-Cl)***

White pellets, m.p. = 44-45 °C, yield 93%. IR (*υ*, cm^-1^): 3050 (C-H ar), 2910 (C-H al), 1700 (C=O), 1615 (C=N), 1565 (C=C); ^1^H NMR (400 MHz, DMSO-*d_6_*): δ_H_ = 8.11 (1H, s, Ar-**H**), 8.00-7.91 (2H, m, Ar-**H**), 7.82-7.77 (2H, m, Ar-**H**), 7.50-7.30 (2H, m, Ar-**H**), 2.52 (3H, s, C**H**_3_ overlapped with DMSO-*d_6_*); ^13^C NMR (125 MHz, DMSO-d6) δ_C_: 167.6 (C=O), 133.2, 131.9, 131.7, 131.2, 131.1, 127.8 (Ar-**C**). Molecular formula is C_12_O_2_N_2_H_10_Cl_2_ with molecular weight = 285 g/ mol, In Mass spectrometry = 283.03210 m/z.

- ***Characterization of 1-acetyl-3-(3,4,5-trimethoxybenzoyl)-1H-imidazol-3-ium chloride (ACIM-OMe)***

White pellets, m.p. = 67-69 °C, yield 91%. IR (*υ*, cm^-1^): 3080 (C-H ar), 2975 (C-H al), 1715 (C=O), 1625 (C=N), 1560 (C=C). ^1^H NMR (400 MHz, DMSO-*d_6_*): δ_H_ = 8.37-8.30 (2H, m, Ar-**H**), 8.13-8.09 (1H, m, Ar-**H**), 7.19 (2H, s, Ar-**H**), 3.78 (6H, s, 2 x OC**H**_3_), 3.68 (3H, s, OC**H**_3_), 2.50 (3H, s, C**H**_3_ overlapped with DMSO-*d_6_*). Molecular formula is C_15_O_5_N_2_H_17_Cl with molecular weight = 340.5 g/ mol, In Mass spectrometry = 338.06540 m/z.

- ***Characterization of 1-acetyl-3-(4-nitrobenzoyl)-1H-imidazol-3-ium chloride (ACIM-NO_2_)***

White pellets, m.p. 56-58 °C, yield 93%. IR (*υ*, cm^-1^): 3035 (C-H ar), 2945 (C-H al), 1710 (C=O), 1610 (C=N), 1555 (C=C). ^1^H NMR (400 MHz, DMSO-*d_6_*): δ_H_ = 8.36-8.32 (2H, m, Ar-**H**), 8.15-8.12 (2H, m, Ar-**H**), 7.76-7.67 (2H, m, Ar-**H**), 7.29-7.26 (1H, m, Ar-**H**), 2.41 (3H, s, C**H**_3_); ^13^C NMR (125 MHz, DMSO-d6) δ_C_: 166.8 (C=O), 150.3, 137.2, 134.2, 131.1, 124.1, 119.7 (Ar-**C**). Molecular formula is C_12_O_4_N_3_H_10_Cl with molecular weight = 295.5 g/ mol, In Mass spectrometry 294.80680 m/z.

**Sup. Fig 1**: FT-IR spectrum of compound ***ACIM-H***.

**Sup. Fig 2**: FT-IR spectrum of compound ***ACIM-OMe***.

**Sup. Fig 3**: FT-IR spectrum of compound ***ACIM-Cl***.

**Sup. Fig 4**: FT-IR spectrum of compound ***ACIM-NO_2_***.


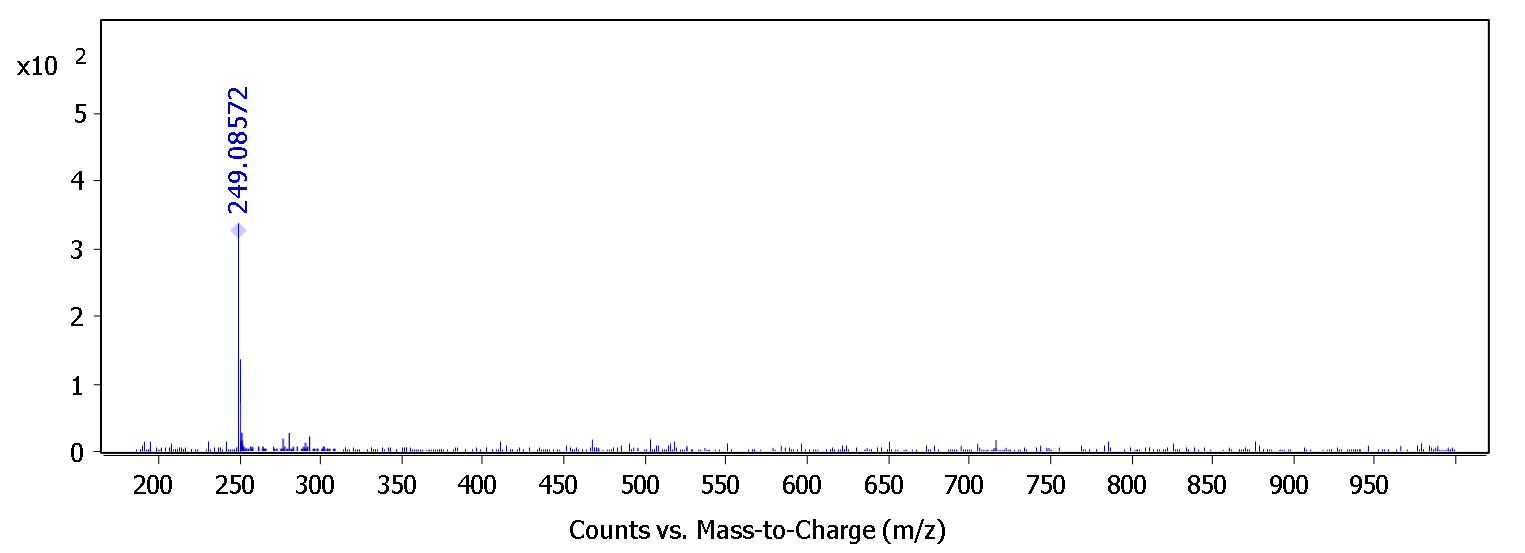


**Sup. Fig 5**: Mass spectrometry of compound ***ACIM-H***.


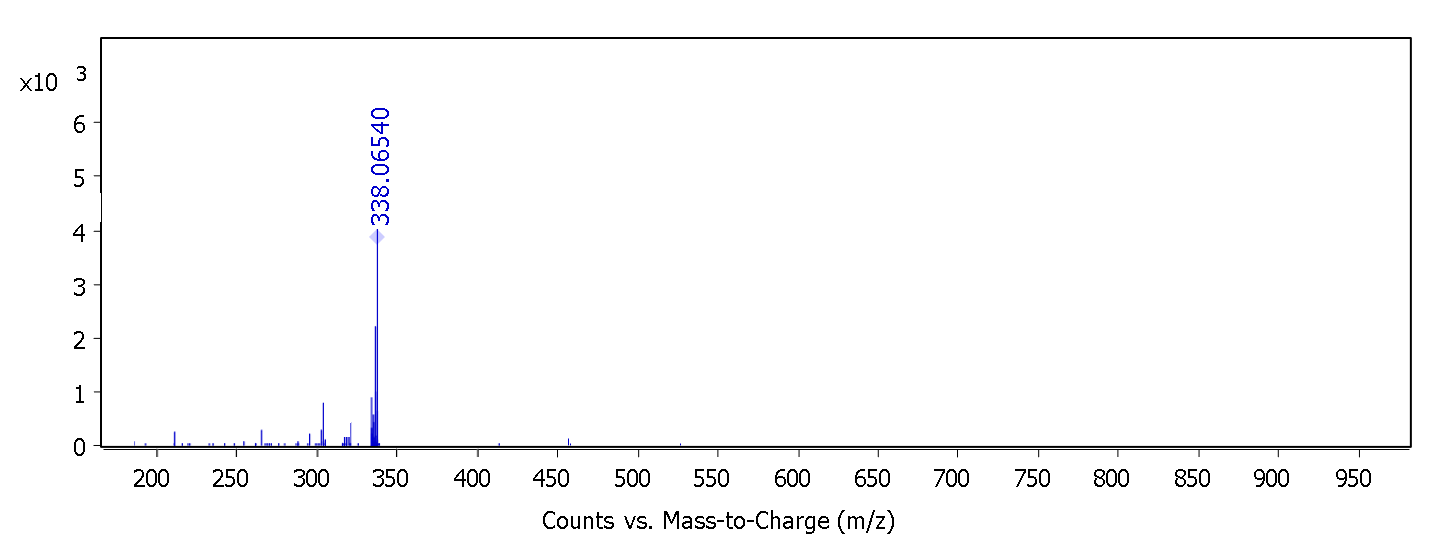


**Sup. Fig 6**: Mass spectrometry of compound ***ACIM-OMe***.


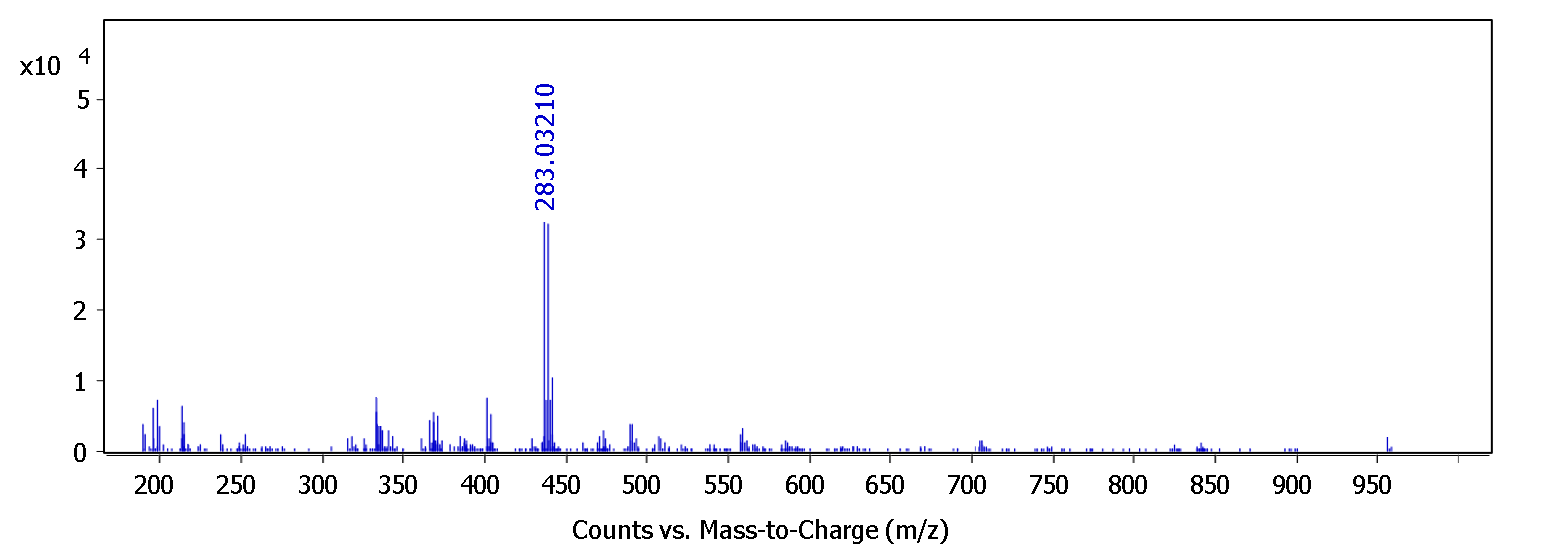


**Sup. Fig 7**: Mass spectrometry compound ***ACIM-Cl***.


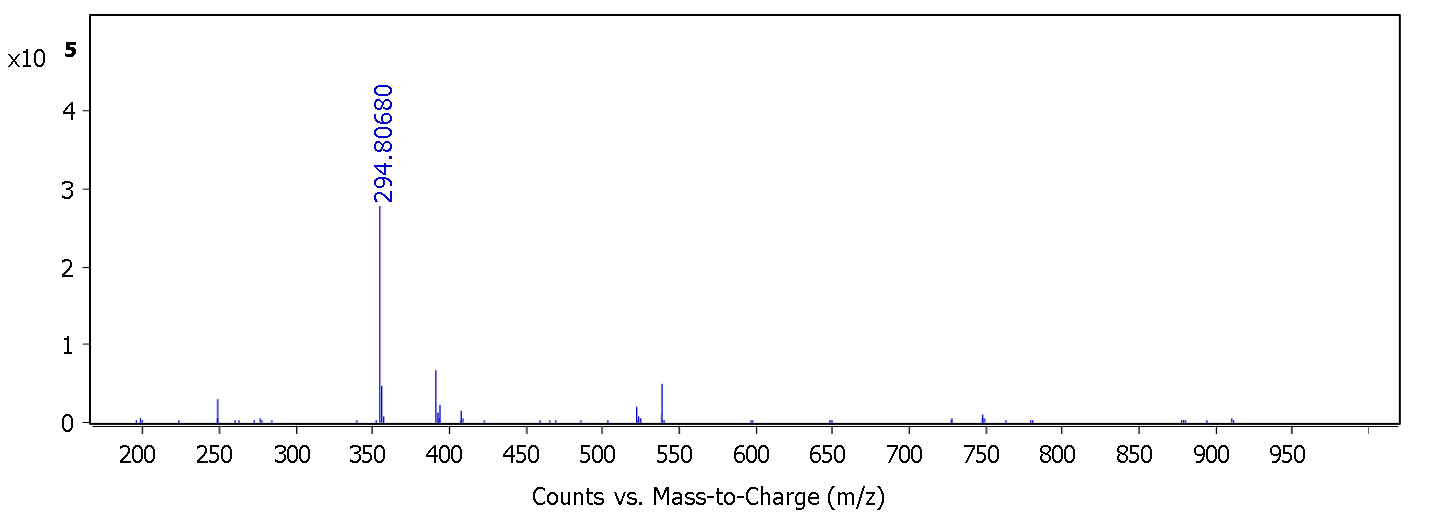


**Sup. Fig 8**: Mass spectrometry compound ***ACIM- NO_2_***.


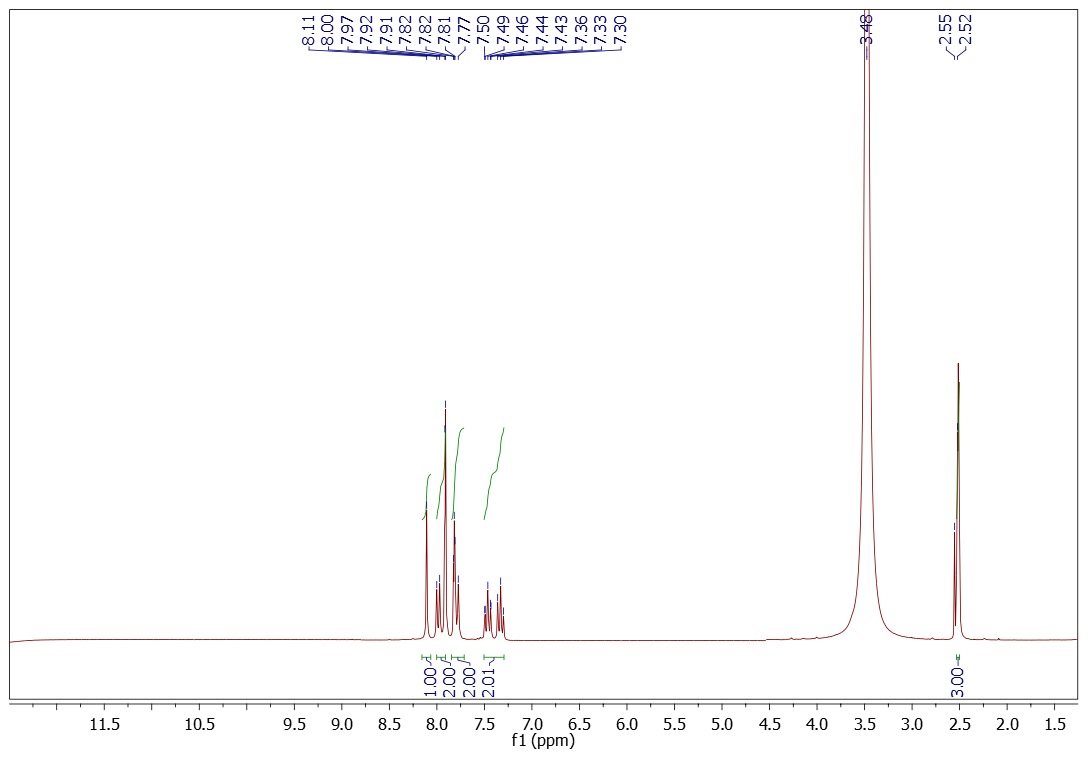


**Sup. Fig 9**: ^1^H NMR spectrum of compound ***ACIM-Cl***.

**Sup. Fig 10**: C^13^ NMR spectrum of compound ***ACIM-Cl***.


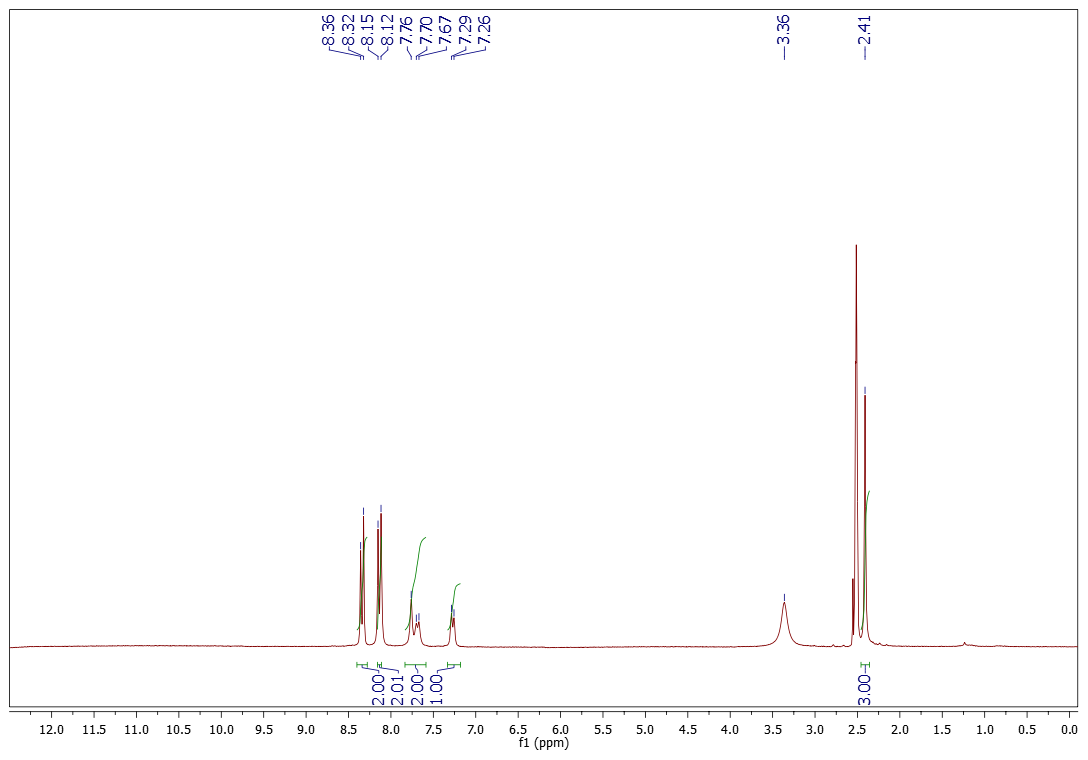


**Sup. Fig 11**: ^1^H NMR spectrum of compound ***ACIM-NO_2_***.

**Sup. Fig 12**: C^13^ NMR spectrum of compound ***ACIM-NO_2_***.

**Sup. Fig 13**: Polarization curve of the tested CS: at different concentrations for **ACIM-H** at 298 K.

**Sup. Fig 14**: Polarization curve of the tested CS: at different concentrations for **ACIM-Cl** at 298 K.

**Sup. Fig 15**: Polarization curve of the tested CS: at different concentrations for **ACIM-NO_2_** at 298 K.

**Sup. Fig 16**: Polarization curve of the tested CS: at temperatures for concentration 20.58 x 10^-5^ M for **ACIM-H*.***

**Sup. Fig 17**: Polarization curve of the tested CS: at temperatures for concentration 20.58 x 10^-5^ M for **ACIM-Cl.**

**Sup. Fig 18**: Polarization curve of the tested CS: at temperatures for concentration 20.58 x 10^-5^ M for **ACIM-NO_2_.**

**Sup. Fig 19**: El-Awady model for different concentrations and temperatures for **ACIM-H**.

**Sup. Fig 20**: El-Awady model for different concentrations and temperatures for **ACIM-Cl**.

**Sup. Fig 21**: El-Awady model for different concentrations and temperatures for **ACIM-NO_2_**.

**Sup. Fig 22**: Free energy vs T to calculate Enthalpy and Entropy for **ACIM**.

**Sup. Fig 23**: Arrhenius diagrams for calculating activation energy based on the relationship between temperature and limiting current with and without **ACIM-H**.

.

**Sup. Fig 24**: Arrhenius diagrams for calculating activation energy based on the relationship between temperature and limiting current with and without **ACIM-Cl**.

**Sup. Fig 25**: Arrhenius diagrams for calculating activation energy based on the relationship between temperature and limiting current with and without **ACIM-NO_2_**.

**Sup. Table 1:** The equations are used to compute the additional parameters for **ACIM**.

| **I =** $\mathbf{-}$ **E_HOMO_** | **A =** $\mathbf{-}$ **E_LUMO_** | **X =**$\frac{\mathbf{(I}\boldsymbol{+}\mathbf{A)}}{\boldsymbol{2}}$ |
| --- | --- | --- |
| **μ =** $\mathbf{-}$ **X** | **ƞ =** $\frac{\mathbf{(I} \mathbf{- A)}}{\boldsymbol{2}}$ | **σ =**$\frac{\boldsymbol{1}}{\mathbf{ƞ}}$ |
| **∆E_b.d_** $\mathbf{=}\frac{\mathbf{-ƞ}}{\boldsymbol{4}}$ | **ω =** $\frac{\boldsymbol{\mu}^{\boldsymbol{2}}}{\mathbf{2ƞ}}$ | **ω^-^ =** $\frac{\mathbf{(3I+A)}^{\mathbf{2}}}{\mathbf{16(I-A)}}$ |
| **ω^+^ =** $\frac{\mathbf{(I+3A)}^{\mathbf{2}}}{\mathbf{16(I-A)}}$ | **∆ω^±^ = (ω^+^- (-ω^-^))** | **∆ω^±^ = (ω^+^-** $\frac{\mathbf{1}}{\boldsymbol{\omega}^{\mathbf{-}}}$**)** |
| **∆N =** $\frac{\left( \mathbf{X}_{\mathbf{Fe}}\mathbf{- X} \right)}{\mathbf{2}\left( \mathbf{ƞ}_{\mathbf{Fe}}\mathbf{+ ƞ} \right)}$ | **∆E_steel/inh_ =** $\frac{\left( \mathbf{X}_{\mathbf{Fe}}\mathbf{-}\mathbf{X}_{\mathbf{inh}} \right)^{\boldsymbol{2}}}{\boldsymbol{4}\left( \mathbf{ƞ}_{\mathbf{Fe}}\mathbf{+}\mathbf{ƞ}_{\boldsymbol{inh}} \right)}$ | |

**Sup. Table 2:** Fukui indices, local dual descriptors, and Mulliken charges of various atoms in **ACIM-OMe** in the gaseous state.

**Sup. Table 3:** Fukui indices, local dual descriptors, and Mulliken charges of various atoms in **ACIM-OMe** in the aqueous state.

**Sup. Table 4:** The equations are used to calculate Fukui indices and local dual descriptors parameters for **ACIM-OMe**.

| f_k_^+^ = ρ_k_ (N+1) $-$ ρ_k_ (N) (nucleophilic attack) |
| --- |
| f_k_^-^ = ρ_k_ (N) $-$ ρ_k_ (N-1) (electrophilic attack) |
| ∆f = (f_k_^+^) $-$ (f_k_^-^) |
| ∆ σ = (σ_k_^+^) $-$ (σ_k_^-^) |
| ∆ ω = (ω_k_^+^) $-$ (ω_k_^-^) |
